# Supplementary material for: The efficacy of Netrod Six-electrode Radiofrequency Renal Denervation system: mathematical modeling, phantom experiments and animal studies
Source: PLoS One. 2025 May 27;20(5):e0323336. doi: 10.1371/journal.pone.0323336 (PMC12111292; doi:10.1371/journal.pone.0323336)
Supplement: Attachment — Submitted filename: PONE-D-24-46135_R1_reviewer.pdf [file pone.0323336.s002.pdf]

# PLOS ONE

## Study on the efficacy of NetrodTM Six-electrode Radiofrequency Renal Denervation system on renal artery vessels

--Manuscript Draft--

| | |
|---|---|
| **Manuscript Number:** | PONE-D-24-46135R1 |
| **Article Type:** | Research Article |
| **Full Title:** | Study on the efficacy of NetrodTM Six-electrode Radiofrequency Renal Denervation system on renal artery vessels |
| **Short Title:** | NetrodTM Six-electrode Radiofrequency Renal Denervation system |
| **Corresponding Author:** | Qun Nan<br>Beijing University of Technology<br>BEIJING, CHINA |
| **Keywords:** | Radio Frequency Ablation;  Renal Denervation;  Resistant hypertension;  Temperature controlled;  NetrodTM Six-electrode |
| **Abstract:** | The principle of NetrodTM Renal Denervation RF Generator is based on the heating of radiofrequency electrodes to achieve the therapeutic temperature for peripheral nerve ablation with minimal impact on intra-arterial blood flow. The extravascular heat distribution and continuous ablation were evaluated using COMSOL Multiphysics with an idealized 3D model and validated in ex vivo experiment. The safety of ablation was verified by H&E staining of hypertensive dog model in treatment group of animal experiments, and changes in blood pressure before and after the procedure were used to characterize the efficacy. The simulation results showed that the maximum injury depth range of the NetrodTM Six-electrode is 5.08mm and the depth of the ablation zone obtained in the Phantom experiment was 3-6mm. Circumferential coverage was up to 91.99% and the ablation zone was continuously. In animal experiments, eight animals in the treatment group had significantly lower blood pressure (88.9%). Nerve cells in nerve bundles show vacuolar changes and thickening of peripheral sheaths after peripheral nerve ablation of renal arteries.The NetrodTM Six-electrode presented here has potential to effectively and safely ablate renal nerves. Systematic ablation can reach the target temperature in a shorter period of time, with faster warming, greater peripheral coverage of the lesion, and a more continuous ablation zone. |
| **Order of Authors:** | xiaohua Song |
| | jiulin Guo |
| | hongguang Cao |
| | qun Nan |
| **Response to Reviewers:** | Journal Requirements:<br>1. Please ensure that your manuscript meets PLOS ONE's style requirements, including those for file naming.<br>Response: I have changed the format.<br><br>2.Please note that PLOS ONE has specific guidelines on code sharing for submissions in which author-generated code underpins the findings in the manuscript. In these cases, we expect all author-generated code to be made available without restrictions upon publication of the work.<br>Response:Yes, code will be made available upon publication of the work.<br><br>3.Thank you for stating the following financial disclosure:"This work was supported by the National Natural Science Foundation of China [31771021, 11832003]."Please state what role the funders took in the study.  If the funders had no role, please state: ""The funders had no role in study design, data collection and analysis, decision to publish, or preparation of the manuscript."<br>Response: This work was supported by the National Natural Science Foundation of China [31771021, 11832003]. The funder had role in study design, data collection and analysis. |

Powered by Editorial Manager® and ProduXion Manager® from Aries Systems Corporation

4. We note that your Data Availability Statement is currently as follows: All relevant data are within the manuscript and its Supporting Information files.
Response: Data Availability Statement is revised as follows: All relevant data are within the manuscript and its Supporting Information files.

5. Please amend either the abstract on the online submission form (via Edit Submission) or the abstract in the manuscript so that they are identical.
Response: I have modified the abstract to be consistent .

6. Please ensure that you refer to Figure 5 in your text as, if accepted, production will need this reference to link the reader to the figure.
Response: Figure 5 was drawn by us.

Additional Editor Comments:
Additionally, in the first paragraph of Introduction, I would mention the clinical utility of renal denervation currently, beyond hypertension. You could refer to reviews encapsulating these findings, such as: https://doi.org/10.1016/j.cpcardiol.2023.102196.
Response: Thanks for the heads up on that.I added to the introduction the current state of research in hypertension other other clinical effects of renal denervation, etc.And cited the article as reference [11].
Content: Other studies[11] have also shown that RDN has the potential to extend to other autonomic nervous system disorders such as atrial fibrillation[12,13], heart failure[14,15] and tachycardia[16].

Furthermore, the discussion requires work. An additional paragraph on comparing and contrasting your findings with existing literature, what's novel about your results, and some background with pros and cons on all three types of electrodes used and their comparisons in either the introduction or second paragraph of the discussion is warranted.
Response: Thank for your advice, I have made overall revisions to the content of the article. I focus the article on the simulation and experimental data of NetrodTM Six-electrode to verify the safety and effectiveness of this electrode through animal experimental data, and no longer compare the advantages and disadvantages with other types. Therefore, I reduced the description of other types of electrodes.

Review Comments to the Author
Reviewer 1:
1.In this manuscript, the authors examined the depth and circumference of ablation lesions produced by the Netrod and other radiofrequency renal denervation systems through simulation models and phantom experiments. While the information provided has clinical significance, there are several methodological limitations and content inconsistencies that cast doubt on the conclusions.
Response: Thank for your advice, in general I modified my research to focus on the experiments on this electrode. In this article, I have further refined and perfected the data from the animal study, and comprehensively demonstrated the efficacy and safety of the NetrodTM Six-electrode by using the data obtained from simulation, phantom experiments and the animal study. For details of the animal study, please refer to the section labeled “Animal study” (highlighted in red here), which records and analyzes the data (blood pressure changes and histological analysis) from the animal study in detail.

2.The title (referring to efficacy and safety, as shown on the first page, which differs from the title on the first page of the text) is inconsistent with the content presented, which only addresses the extent of ablations and does not discuss efficacy or safety.
Major limitations requiring clarification:
Response: Thank for your suggestion to add animal testing to verify its safety and efficacy. In the course of the experiment, through the monitoring of the animals' blood pressure, the stable performance of the NetrodTM Six-electrode in practical application was clearly demonstrated. Specifically, the experimental animals did not experience any abnormal physiological reactions or adverse reactions after receiving the electrode treatment, and all vital signs remained stable, which fully demonstrates that its safety can stand the test.The details are as follows:
Content: During the postoperative observation period, the experimental animals did not show any abnormalities related to the treatment. No abnormalities such as stenosis or

dissection were observed in the renal arteries of the animals when the angiography was performed before dissection, and no abnormalities were observed when the animals were dissected for observation and sampling. The above experiments prove the safety of the device.
3.Abstract Inconsistency: The abstract on the first page of the PDF file mentions animal studies and histological analysis. However, the main text and its abstract only present data on ablation lesion depth and circumference from different radiofrequency renal denervation systems. Additionally, the titles differ between sections. The content is limited and inconsistent with the title on the first page ("Study on the efficacy and safety of Netrod™ Six-electrode Radiofrequency Renal Denervation system on renal artery vessels"), as no information regarding clinical efficacy and safety is presented.
Response:Thank for your advice. I have refined the animal studies and histological analysis in the main text to validate the study on the efficacy of NetrodTM Six-electrode Radiofrequency through blood pressure changes in the animals and The results of H&E staining of the ablation sites. Renal Denervation system so that the abstract corresponds to the main text.At the same time I improved some of the article's headings to align them with the body content.

4..Unsupported Assumptions in Introduction: The authors assume that "continuous circumferential (ring)" ablation is superior for renal denervation without providing supporting evidence. The Medtronic Symplicity Spyral system deliberately uses a "non-continuous" circumferential ablation pattern to prevent continuous vessel injury, which might lead to renal artery stenosis. The authors should provide histological evidence demonstrating the safety of continuous circumferential ablations achieved by the Netrod before asserting its superiority over non-continuous circumferential ablation in terms of efficacy or safety.
Response:As the reviewer noted, we do not discount the safety of Medtronic products in terms of continuous ablation, but only assessed the safety of continuous ablation with our own ablation electrodes in 18 animals. As a measure of efficacy, blood pressure was measured at regular intervals before and during the post-test observation period, and we measured the change in blood pressure at three points of time after RDN. During survival, animals were observed clinically. To investigate the short- term, medium- term, and long-term safety of the test article, three points of time, 7, 30, and 60 days, were included in this experiment. On postoperative days 7 (±1), 30 (±3) and 60 (±5) (with the day of surgery as 0 day), renal arteriograms were performed in animals scheduled for dissection to visualize the test site and renal arteries with nerves were harvested for immuno-histochemical evaluation. Quantification of protein denaturation and necrosis of renal sympathetic nerves around renal arteries were using hematoxylin-eosin (H&E) staining. Specific experimental results are shown in Animal experiment results. We believe that the design of the net basket electrodes allows multiple electrodes to contact the vessel simultaneously and create a continuous line of ablation at the desired location, which can improve the therapeutic efficacy.

5.Missing Table: Table 3 is referenced in Results 3.1 but is not present in the manuscript.
Response:Yes, thanks for the advice. The tables have been switched in content and location, and all tables have been sorted.

6.Incomplete Information in Figure 9: The vessel diameter in the simulation model should be specified, as this parameter significantly influences the continuity of ablation lesions across different systems.
Response:Yes, thanks for the advice. Figure 9 was removed because the focus was no longer on the helical electrode approach data. The diameter of the vessels in the simulation and in the soma experiments was 6 mm.There is a reference in “Finite element method modeling”:The tissue included two major parts, the renal artery ( 6mm diameter).

7.Inequitable System Comparisons: The default ablation times for different renal denervation systems are either 60 or 120 seconds. Comparing ablation extent at 30 seconds (Figure 8) or 5 seconds (Figure 9) provides an unfair comparison. Results should be shown for at least 60-second ablations across all systems.
Response:Yes, thanks for the advice. I agree with the reviewers that there was unfairness in the way the previous comparisons with other systems were made.

Powered by Editorial Manager® and ProduXion Manager® from Aries Systems Corporation

Therefore, in this revision of the paper, instead of comparing other systems to highlight the advantages of the NetrodTM Six-electrode, we will focus on the presentation of the experimental data of the system itself. We will analyze the data obtained from simulations and in vivo experiments, and through detailed interpretation and analysis of these data, we will strongly argue efficacy and safety of the NetrodTM Six-electrode.

8.Contradictory Results: In Results 3.3, the authors note that phantom experiments allow for optimal manual positioning of the Netrod™ Six-electrode against the vessel wall, while simulation experiments show poorer wall apposition. However, the smaller ablation depth and extent observed in phantom experiments contradict the expected benefits of better contact. This discrepancy requires explanation.
Response:Thanks for the advice. It may be that our expression is not precise enough, we think: the simulation considers rigid walls or does not consider deformation, so the fit is worse than that of the phantom experiments , and therefore the depth is smaller. During the actual operation of the phantom experiments , adjustments can be made manually to make the mesh basket fit tightly, so the fit is better in phantom experiments, and the ablation depth is greater This may be the reason for the difference.

9.Incomplete Methodology: The ablation settings for other renal denervation systems are not described. These parameters should be clearly detailed to substantiate the fairness of the comparisons.
Response:Thanks for the advice. I agree with the reviewers that the previous comparisons with other systems were indeed unfair. Therefore, in this article, we will no longer use the comparison with other systems to highlight the advantages of theNetrodTM Six-electrode, but focus on the results of the experimental data of this system, with the help of concrete experimental data to strongly argue the rationality and effectiveness of this system, so that its advantages can be presented visually and more convincing.

Reviewer 2:
1.The title of the manuscript does not indicate that this is an experiment and nor a clinical study.This is not even an animal study, but a mathematical simulation on software and phantoms that limits the overall relevance of the experiments.This should be specified in the title and the conclusions of the manuscript.
Response:Yes, thank for your advice, and as a whole I've revised my research. The article was refined, mainly because the previous study was biased towards the mathematical simulation of the simulation and phantom experiments, but because of the lack of experimental data from animals or human beings, it limited the overall relevance of the study and did not well reflect the safety feasibility of the studied system, so the study increased the animal studies to further improve the experimental data on the basis of the mathematical simulation and match the simulation data to improve the study of this system.

2.First of all, this experimental study has not been performed in renal arteries, but in a cylindrical synthetic mold made of Polyacrylamide. Therefore, the title should not mention the words renal artery vessels but rather “polyacrylamide phantom”. Likewise, not been performed in biological tissues of living organisms it cannot refer to “safety”.Introduction. Authors should remove the comment on Flex and limit to spiral since the flex has no possibilities of spatial orientation of the electrode.
Response:Yes, thanks for the advice. I added animal testing to the study to demonstrate “safety” through changes in animal blood pressure data and histology.We did animal experiments, and the significant decrease in blood pressure in the animals after surgery and the histologic data after autopsy verified the effectiveness and safety (see Animal experiment results for details). And remove the comment on Flex and limit to spiral since the flex has no possibilities of spatial orientation of the electrode.

3.The NetrodTM Six-electrode device demonstrated effective ablation of renal sympathetic nerves, achieving temperatures of up to 45°C at distances of approximately 5.08 mm (>4 mm) from the electrodes. This confirms the feasibility and efficacy of using the NetrodTM Six-electrode for radiofrequency ablation of the renal sympathetic nerve”.Indeed, such a sentence cannot be accepted since by no means the authors are testing the effect of the device on renal nerves, but in a phantom. The reader would be mislead with such a sentence that should clearly state that was

Powered by Editorial Manager® and ProduXion Manager® from Aries Systems Corporation

changes color as an indirect indicator of heat is a synthetic material and not real tissues.
Response:Yes, thanks for the advice. I think the reviewer made a reasonable point, and this formulation has been deleted, and to add in Phantom experiment validation:"The body mold changes color from blue to white when the temperature exceeds 45°C."

4.To affirm that the observed changes obtained after heating the RF electrodes are effective in ablating nerves, the experiments should be performed in animal models or in human cadavers.
Response:Yes, thanks for the advice. The study has added animal experiments. Comparisons were made with the simulation to illustrate the accuracy of the simulation. Also blood pressure changes by animal experiments and renal arteriography was performed on animals scheduled for dissection to visualize the test site and renal arteries with nerves were collected for immunohistochemical evaluation.

5.Similarly, further in the results: "NetrodTM Six-electrode to be positioned optimally against the vessel wall"Or in the discussion: "Results show that the NetrodTM Six-electrode system provides more comprehensive coverage and better continuity of the ablation zone compared to both the single electrode and the spiral six electrodes under the same setup. Lesion formation is influenced by several factors, including ablation power, ablation time, the number of electrodes, the area in contact with tissue, and blood flow"
Sentences as such cannot be proposed since there is no vessel wall, but a simple synthetic cylinder. Authors should therefore be clear with the reader and avoid any reference to tissues, vessels, blood and nerves.
Response:Yes, thank you for your suggestion. Instead of comparing the ablation zones of the two, we did a complete simulation, ex vivo and animal experiments for this electrode. So this part has been deleted.

6.Comparison of ablation effect of NetrodTM Six-electrode with single electrode and spiral six electrode: the authors do not explain the method used to detect and quantify the dispersion of the heath in figures 7,8 and 9.
Response:Yes, thanks for the advice. We will no longer compare the ablation zones of the different electrodes. Figure 7.8.9 has been changed so that the above issue no longer exists.

7.What is the model used to test 6 spiral electrodes? The commercially available spiral system (Medtronic) has 4 electrodes and not 6. If the spiral 6 electrodes used in this study was developed by the investigators please provide details of the model used and clarify that is not the one commercially available, and that the effect described for 6 electrodes may not apply to a 4 electrodes spiral catheter.
Response:Yes, thanks for the advice. Since the spiral electrode studied at the beginning of the article was based on the spiral electrode simulation of the spiral construction setup, without using the commercially available system, the study is not too rigorous, so the spiral system is no longer studied in this article, including the relevant simulation data.

8.Figure 10. Please explain how the phantom model was constructed and how was the effect of RF assessed.
Response:Yes, thanks for the advice. The model construction and materials are described in the Phantom experiment validation section. This model was used to assess the ablation depth of the device and the continuity of the ablation zone by the change in tissue color to validate the accuracy of the data from the simulation experiment.

9.The authors affirm that "The electrodes are treated as uniformly heated sources, spreading heat consistently into the bloodstream and surrounding tissue through conduction" how can they say that if their model has no fluid circulation, and therefore, temperature dispersion by blood temperature and flow cannot be calculated?
Response:Yes, thank you for your advice. The simulation takes into account the effect of blood flow, so there is fluid circulation, and it is mentioned in the text that "The arterial inlet flow rate was set to 0.5 m/s, and the ablation time was set to

| | |
|---|---|
| | 120s."However, fluid circulation was not considered in the phantom due to the material structure and the specificity of the experiment.<br><br>10.Authors used 60°C for 120 seconds with their device.Other commercially available use variable temperature for 60 seconds. The authors should explain on which observations (ideally obtained in animals) they base the selection of temperature and duration of the energy delivery, and demonstrate in biological tissue of animals or cadavers, that such combination of temperature and duration is not creating unintended tissue damage to neighbor structures such as the artery wall, veins, lymphatic or urinary structures.<br>Response:Yes, thanks for the advice. The working time of the device is adjustable, and for the time case we added animal experiments, which further verified the safety and effecty of ablation at this time by animal experimental data (ablation time 120s, temperature control setting 60°C). It is not creating unintended tissue damage to neighbor structures such as the artery wall, veins, lymphatic or urinary structures.<br>Content:The degree of local inflammation and other pathological reactions in the treatment group was significantly greater than that in the blank control group at all points of time, which was considered to be caused by intraoperative ablation damage to tissues, but did not show significant safety problems, and all of them decreased with the prolongation of postoperative time. |
| **Additional Information:** | |
| **Question** | **Response** |
| **Financial Disclosure**<br><br>Enter a financial disclosure statement that describes the sources of funding for the work included in this submission. Review the submission guidelines for detailed requirements. View published research articles from *PLOS ONE* for specific examples.<br><br>This statement is required for submission and **will appear in the published article** if the submission is accepted. Please make sure it is accurate.<br><br>**Funded studies**<br>Enter a statement with the following details:<br>• Initials of the authors who received each award<br>• Grant numbers awarded to each author<br>• The full name of each funder<br>• URL of each funder website<br>• Did the sponsors or funders play any role in the study design, data collection and analysis, decision to publish, or preparation of the manuscript?<br><br>Did you receive funding for this work? | Yes |
| Please add funding details. | This work was supported by the National Natural Science Foundation of China |

| | |
|---|---|
| as follow-up to "**Financial Disclosure**<br><br>Enter a financial disclosure statement that describes the sources of funding for the work included in this submission. Review the submission guidelines for detailed requirements. View published research articles from *PLOS ONE* for specific examples.<br><br>This statement is required for submission and **will appear in the published article** if the submission is accepted. Please make sure it is accurate.<br><br>**Funded studies**<br>Enter a statement with the following details:<br>• Initials of the authors who received each award<br>• Grant numbers awarded to each author<br>• The full name of each funder<br>• URL of each funder website<br>• Did the sponsors or funders play any role in the study design, data collection and analysis, decision to publish, or preparation of the manuscript?<br><br>Did you receive funding for this work?" | [31771021, 11832003]. The funder had role in study design, data collection and analysis. |
| Please select the country of your main research funder (please select carefully as in some cases this is used in fee calculation).<br>as follow-up to "**Financial Disclosure**<br><br>Enter a financial disclosure statement that describes the sources of funding for the work included in this submission. Review the submission guidelines for detailed requirements. View published research articles from *PLOS ONE* for specific examples.<br><br>This statement is required for submission and **will appear in the published article** if the submission is accepted. Please make sure it is accurate. | CHINA - CN |

| | |
|---|---|
| **Funded studies**<br>Enter a statement with the following details:<br>• Initials of the authors who received each award<br>• Grant numbers awarded to each author<br>• The full name of each funder<br>• URL of each funder website<br>• Did the sponsors or funders play any role in the study design, data collection and analysis, decision to publish, or preparation of the manuscript?<br><br>Did you receive funding for this work?" | |
| **Competing Interests**<br><br>Use the instructions below to enter a competing interest statement for this submission. On behalf of all authors, disclose any competing interests that could be perceived to bias this work—acknowledging all financial support and any other relevant financial or non-financial competing interests.<br><br>This statement is required for submission and **will appear in the published article** if the submission is accepted. Please make sure it is accurate and that any funding sources listed in your Funding Information later in the submission form are also declared in your Financial Disclosure statement.<br><br>View published research articles from *PLOS ONE* for specific examples. | The authors have declared that no competing interests exist. |

| | |
|---|---|
| **NO authors have competing interests**<br><br>Enter: *The authors have declared that no competing interests exist.*<br><br>**Authors with competing interests**<br><br>Enter competing interest details beginning with this statement:<br><br>*I have read the journal's policy and the authors of this manuscript have the following competing interests: [insert competing interests here]*<br><br>* typeset | |
| **Ethics Statement**<br><br>Enter an ethics statement for this submission. This statement is required if the study involved:<br><br>• Human participants<br>• Human specimens or tissue<br>• Vertebrate animals or cephalopods<br>• Vertebrate embryos or tissues<br>• Field research<br><br>Write "N/A" if the submission does not require an ethics statement.<br><br>General guidance is provided below. Consult the submission guidelines for detailed instructions. **Make sure that all information entered here is included in the Methods section of the manuscript.** | Dogs subjected to animal testing and experimental protocols were treated in strict accordance approved by the Institutional Animal Care Committee of Shanghai Harborside Medical Technology Co., Ltd., based on the Institutional Animal Care and Use Committees（IACUC）Animal Care and Use Program on Animal Experimentation (approval No.IACUC-2021-003). |

Powered by Editorial Manager® and ProduXion Manager® from Aries Systems Corporation

| | |
|---|---|
| **Format for specific study types**<br><br>**Human Subject Research (involving human participants and/or tissue)**<br>• Give the name of the institutional review board or ethics committee that approved the study<br>• Include the approval number and/or a statement indicating approval of this research<br>• Indicate the form of consent obtained (written/oral) or the reason that consent was not obtained (e.g. the data were analyzed anonymously)<br><br>**Animal Research (involving vertebrate animals, embryos or tissues)**<br>• Provide the name of the Institutional Animal Care and Use Committee (IACUC) or other relevant ethics board that reviewed the study protocol, and indicate whether they approved this research or granted a formal waiver of ethical approval<br>• Include an approval number if one was obtained<br>• If the study involved *non-human primates*, add *additional details* about animal welfare and steps taken to ameliorate suffering<br>• If anesthesia, euthanasia, or any kind of animal sacrifice is part of the study, include briefly which substances and/or methods were applied<br><br>**Field Research**<br><br>Include the following details if this study involves the collection of plant, animal, or other materials from a natural setting:<br>• Field permit number<br>• Name of the institution or relevant body that granted permission | |
| **Data Availability**<br><br>Authors are required to make all data underlying the findings described fully available, without restriction, and from the time of publication. PLOS allows rare exceptions to address legal and ethical concerns. See the PLOS Data Policy and FAQ for detailed information. | Yes - all data are fully available without restriction |

| | |
|---|---|
| A Data Availability Statement describing where the data can be found is required at submission. Your answers to this question constitute the Data Availability Statement and **will be published in the article**, if accepted.<br><br>**Important:** Stating 'data available on request from the author' is not sufficient. If your data are only available upon request, select 'No' for the first question and explain your exceptional situation in the text box.<br><br>Do the authors confirm that all data underlying the findings described in their manuscript are fully available without restriction? | |
| **Describe where the data may be found in full sentences. If you are copying our sample text, replace any instances of XXX with the appropriate details.**<br><br>• If the data are **held or will be held in a public repository**, include URLs, accession numbers or DOIs. If this information will only be available after acceptance, indicate this by ticking the box below. For example: *All XXX files are available from the XXX database (accession number(s) XXX, XXX.*).<br>• If the data are all contained **within the manuscript and/or Supporting Information files**, enter the following: *All relevant data are within the manuscript and its Supporting Information files.*<br>• If neither of these applies but you are able to provide **details of access elsewhere**, with or without limitations, please do so. For example:<br><br>*Data cannot be shared publicly because of [XXX]. Data are available from the XXX Institutional Data Access / Ethics Committee (contact via XXX) for researchers who meet the criteria for access to confidential data.*<br><br>*The data underlying the results presented in the study are available from (include the name of the third party* | All relevant data are within the manuscript and its Supporting Information files. |

| | |
|---|---|
| *and contact information or URL).*<br>• This text is appropriate if the data are owned by a third party and authors do not have permission to share the data.<br><br>* typeset | |
| Additional data availability information: | |

Manuscript Click here to access/download;Manuscript;manuscript.docx

# Study on the efficacy of Netrod™ Six-electrode Radiofrequency Renal Denervation system on renal artery vessels

Xiaohua Song[ac1] Jiulin Guo[b1] Hongguang Cao[b] Qun Nan[a*]
[a] College of Chemistry and Life Science, Beijing University of Technology, Beijing, China
[b] Shanghai Golden Leaf MedTech CO., Ltd, Shanghai, China
[c] Industrial Informatization and Science and Technology Bureau of Changqing District, Jinan, China

**Abstract:** The Netrod™ Renal Denervation RF Generator operates on the principle of heating radiofrequency electrodes to reach the therapeutic temperature necessary for peripheral nerve ablation while minimizing the impact on intra-arterial blood flow. Extravascular heat distribution and continuous ablation efficacy were evaluated using COMSOL Multiphysics with an idealized 3D model, and findings were validated through phantom experiments. Simulation results indicated that the maximum ablation depth achievable with the Netrod™ Six-electrode is 5.08 mm, with circumferential coverage reaching up to 91.99%. The depth of the ablation zone measured in the phantom experiments ranged from 4 to 6 mm. The Netrod™ Six-electrode demonstrated significant potential for effective renal nerve ablation. Systematic ablation can attain the target temperature in a shorter time frame, with faster warming, greater peripheral coverage of the lesion, and a more continuous ablation zone.

**Keywords:** Radio Frequency Ablation; Renal Denervation; Resistant hypertension; Temperature controlled; Netrod™ Six-electrode.

## 1 Introduction

Excessive sympathetic activation plays a significant role in the development and maintenance of hypertension [1-3]. Several studies have indicated that blocking sympathetic nerves may serve as a viable target for hypertension treatment [4-6]. Renal denervation (RDN) is a minimally invasive procedure that reduces blood pressure by modulating sympathetic hyperexcitability [7]. This technique is gaining acceptance and widespread application in clinical settings across various countries [8].

Although research related to RDN is still in its early stages, several new devices have been developed in recent years [9]. Common radiofrequency (RF) catheter ablation devices include the Symplicity System, Symplicity Spyral System, EnligHTN System, FlashPoint™ System, and Iberis System. The Symplicity Catheter System (including Symplicity HTN-1 [6], Symplicity HTN-2 [4], and DENERHTN [10]) is the most widely utilized device for RDN. However, it has a notable limitation: the catheter features only a single electrode, which allows for only single-point ablation.

In contrast, Medtronic's Symplicity Spyral System and Shanghai Anton's Iberis System deliver RF energy in a spiral pattern [11, 12]. This spiral ablation technique ensures circumferential coverage, making the procedure easier and reducing procedure time. However, a challenge remains in ensuring that all electrodes maintain contact for continuous ablation.

The EnligHTN System facilitates rapid, efficient, and precise catheterization while minimizing fluoroscopy time and the volume of contrast material used [13]. The FlashPoint™ System [14], on the other hand, is a domestic multi-polar ablation catheter device featuring four pairs of electrodes and

* Corresponding author College of Chemistry and Life Science, Beijing University of Technology, Beijing, 100124, China
E-mail addresses: nanqun@ bjut. edu. cn
[1] Xiaohua Song and Jiulin Guo are Co-first authors

a spiral design at the tip. Notably, the head end of the sheath can be straightened for easier insertion and will revert to a spiral shape upon the removal of external force. This design incorporates a degree of tension, enhancing energy delivery against to the vessel wall, particularly at the bends of the renal artery.

The development of the aforementioned devices emphasizes the importance of achieving rapid, simple, and circumferential ablation with complete apposition. Ring ablation enables the simultaneous treatment of renal sympathetic nerves in a single 360° session. Considering these advancements, Shanghai Golden Leaf MedTech Co., Ltd. proposes the concept of a "Continuous Energy Field," utilizing a net basket electrode design and increasing the number of electrodes.

In the following sections, we will detail the numerical simulations and validation experiments conducted with the Netrod™ Six-electrode. This research aims to provide a scientific basis for assessing the clinical efficacy of renal sympathetic nerve ablation using the Netrod™ Six-electrode.

## 2 Methods

### 2.1 Equipment Introduction

Netrod™ Six-electrode Radiofrequency Renal Denervation system (Shanghai Golden Leaf MedTech Co. Ltd) includes Netrod™ Renal Denervation RF Generator and Netrod™ Six-electrode Radiofrequency Renal Denervation catheter (Figure 1).

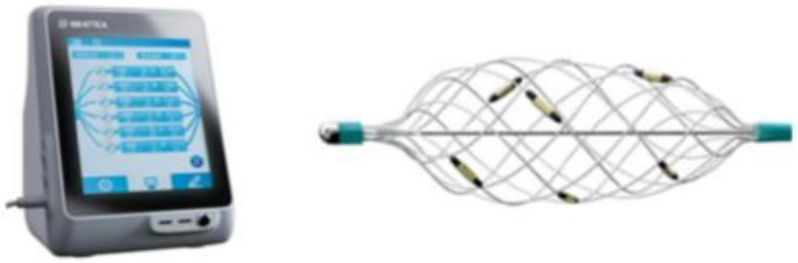

Figure 1 Radiofrequency ablation mainframe and Netrod™ Six-electrode structure diagram

The generator primarily consists of a microcontroller, a signal source circuit, a power drive circuit, a power output circuit, a detection circuit, and a display operation control unit. The detection circuit mainly detects monitors voltage, current, and temperature levels (Figure 2).

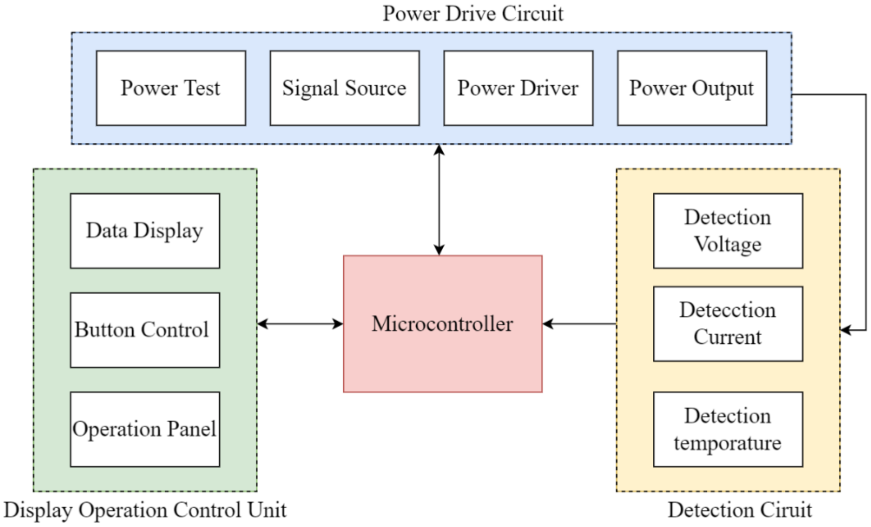


Figure 2 Netrod™ Renal Denervation RF Generator components

The microcontroller is the core component of the generator. It not only processes the voltage,

current, and temperature data transmitted by the detection circuit, but also outputs impedance, temperature, and power data to the display operation control unit. Furthermore, it intelligently adjusts the power output of the signal sources based on temperature and impedance to maintain the electrode surface temperature at a constant value, thereby ensuring safety during operation. The display operation control unit real-time records and displays the temperature, impedance, and power of each electrode during the radiofrequency ablation, and draws relevant curves (Figure 3), allowing medical staff to monitor the working status of the system in real-time through the display.

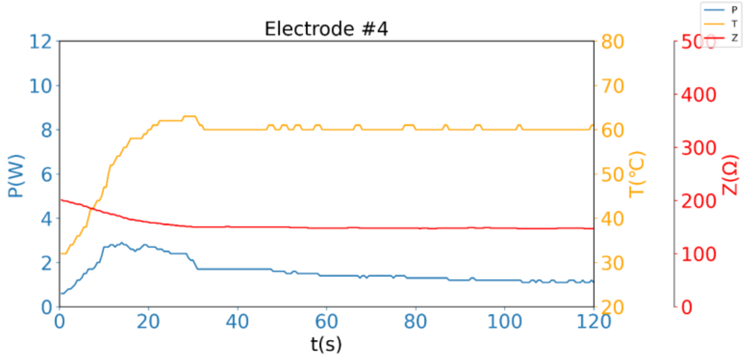

Figure 3 120 seconds of radiofrequency ablation, the power(P), temperature(T), and impedance(Z) of electrode 4 change over time

The radiofrequency ablation catheter is composed of a central wire drawing with a developing head, a mesh tube connector, an electrode, a mesh basket bracket, a catheter sleeve, a seven-hole tube, and a handle assembly (Figure 4).

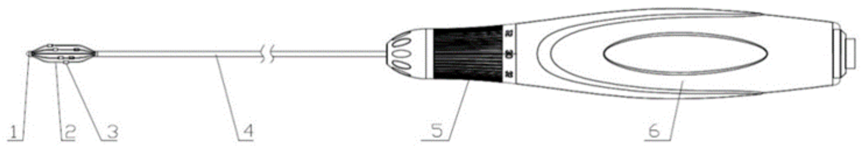

Figure 4 Netrod$^{TM}$ Six-electrode Basket Radiofrequency Renal Denervation Catheter. 1: Central wire drawing with a developing head; 2: Mesh basket bracket; 3: Electrode; 4: Seven-hole tube; 5: Adjustment knob; 6: Handle

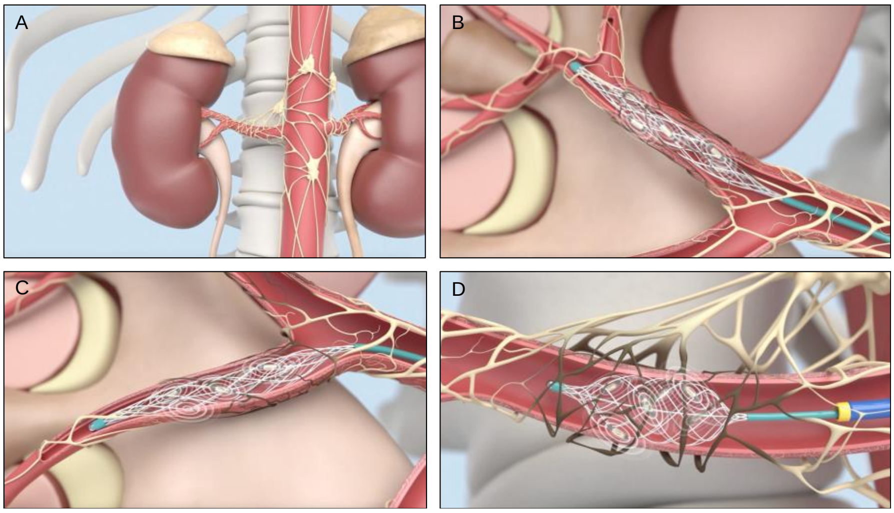

Figure 5 Basket catheter ablation of renal sympathetic nerves. A. Renal sympathetic nerve distribution; B,C. The basket catheter enters the branch for ablation; D. The basket catheter expands the basket stent to perform ablation in the main trunk of the renal artery.

**2.2 Finite element method modeling**

We use COMSOL Multiphysics software (COMSOL, Burlington MA, USA), a finite element simulation software, to perform electromagnetic field and temperature field coupling calculations.

In radiofrequency ablation therapy, thermal energy is generated by converting the electrical energy that is applied as the application of radiofrequency current to the target renal artery tissue. The electric field control equation[15] can be calculated by solving the following Laplace equation. The frequency of the RF current is set to 470 KHz[16] , and the biological medium can be considered resistive because the displacement current is much smaller than the conduction current and thus can be neglected[17]. The distributed heat source Qr is given by Eq. (1)

$$Q_r = J \cdot E \tag{1}$$

Where J is the current density, measured in A/m$^2$; Qr is the distributed heat source, measured in J. The voltage is obtained from the solved Laplace equation, which is the controlling equation for solving the electric field problem in the presence of the internal power supply; see Eq. (2) and (3).

$$\nabla \cdot (\sigma \nabla V) = 0 \tag{2}$$

$$E = -\nabla V \tag{3}$$

Where V is the voltage, measured in V; E is the electric field strength, measured in V/m;σ is the electrical conductivity, measured in S/m。In the model, the boundary conditions at the current interface are set as follows:

$$\begin{cases} V = 0 & \text{All external surfaces of the abdominal cavity} \\ V = V_0 & \text{Six electrodes outer surface} \\ \mathbf{n} \cdot J = 0 & \text{All other domain boundaries} \end{cases} \tag{4}$$

The initial potential is set to 0 V, the outer surface of the electrodes is set to a voltage value with temperature control. In the entire model, all the outer surfaces of the tissue are grounded with zero current density.

In the simulation, an idealized three-dimensional vascular geometry model of the renal artery was constructed, as shown in Figure 6. The tissue included two major parts, the renal artery（6mm diameter) and the abdominal cavity (26mm×26mm×30mm), which was constructed as the external environment for the simulation. The arterial inlet flow rate was set to 0.5 m/s, and the ablation time was set to 120s.

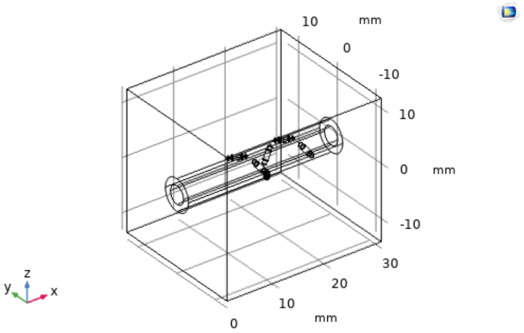


Figure 6 Three-dimensional renal artery tissue and net basket-based RF electrode model

Table 1 shows material property parameters of the abdominal part, blood and electrodes[18-22].

| Material Properties | ρ(kg/m$^3$) | σ (S/m) | C(J/kg·°C) | K(W/(m·°C) | $\xi_r$ |
|---|---|---|---|---|---|
| Blood | 1050 | 0.99 | 4180 | 0.54 | 58.3 |
| Abdominal cavity | 1060 | 1.69 | 3600 | 0.512 | 43.03 |
| Agar | 1300 | 2.3 | 4182 | 0.6 | 57.99 |

| | | | | | |
|---|---|---|---|---|---|
| Gold Electrode | 19300 | 4.1e7 | 127 | 315 | 1 |

Where ρ is the density, $Kg/m^3$; σ is the conductivity, S/m; C is the specific heat capacity, J/kg·°C; K is the thermal conductivity, m·°C; $\xi_r$ is the relative dielectric constant.

**2.3 Phantom experiment validation**

Tissue-mimicking phantoms play a crucial role in the development, performance testing, quality assurance, and formulation of effective treatment plans for RF ablation devices[23]. Given that the walls of real human renal vessels are very thin, the wall thickness is often neglected in experimental models. In this study, we utilized a cylindrical mold with a vascular channel, where the surrounding area simulates human abdominal tissue. The entrance diameter of the vascular channel is 6 mm, corresponding to the dimensions of the simulation model. Polyacrylamide is particularly advantageous due to its stability at high temperatures. Many researchers have reported successful applications of polyacrylamide-based transparent tissue-mimicking molds, incorporating protein as a temperature indicator for thermal ablation studies[24] . The raw materials and formulation details for the specific mold used in this study are presented in Table 2.[25]

Table 1 Proportioning table for the preparation of 100 ml solution[26]

| Substance Name | Weighing |
|---|---|
| Citric acid | 2.49g |
| Sodium citrat | 2.66g |
| Acrylamide | 7.62g |
| Methylene-bisacrylamide | 0.4g |
| Bovine serum protein | 2.0g |
| Degassed water | 100ml |
| Ascorbic acid | 0.1g |
| 1%$FeSO_4$ | 1.0ml |
| 3%$H_2O_2$ | 1.0ml |

The target temperature for the electrode was set at 60˚C, and the experiment was conducted for a duration of 120 seconds. For thermal damage analysis, we evaluated the depth of thermal damage in the body mold through post-experimental assessment. The body mold changes color from blue to white when the temperature exceeds 45°C. Therefore, the color change on the surface of the body mold served as an indicator of the ablation effect.

**3 Results**

**3.1 Simulation results of Netrod™ Six-electrode**

The electrodes are treated as uniformly heated heat sources, spreading heat consistently into the bloodstream and surrounding tissue through conduction. As the ablation time increases, the ablation area expands, with specific data detailed in Table 3.

Table 2 Ablation of electrodes at different times

| | Electrode temperature/˚C | Distance between 45˚C boundary and electrodes/mm |
|---|---|---|
| 5s | 48.6 | 0.23 |
| 30s | 60.0 | 2.69 |
| 60s | 60.0 | 3.78 |
| 120s | 60.0 | 5.08 |

Analysis of the ablation region indicates that the Netrod™ Six-electrode can achieve 360° ablation. With prolonged ablation time, the boundary of the region where the temperature reaches 45°C expands. Since temperature-controlled RF ablation was employed, the maximum temperature was confined to the vicinity of the electrodes, as shown in Table 3. The Netrod™ Six-electrode device demonstrated effective ablation of renal sympathetic nerves, achieving temperatures of up to 45°C at distances of approximately 5.08 mm (>4 mm) from the electrodes. This confirms the feasibility and efficacy of using the Netrod™ Six-electrode for radiofrequency ablation of the renal sympathetic nerve.

### 3.2 Comparison of ablation effect of Netrod$^{TM}$ Six-electrode with single electrode and spiral six electrode

The ablation characteristics of three RF applicators, i.e., balloon-based four electrodes, four spiral electrodes, and single electrode systems, were compared in previous study[27]. Based on previous work, we construct an effective model for the proposed Netrod$^{TM}$ Six-electrode and compared it with single electrode, and spiral electrode simulation results.

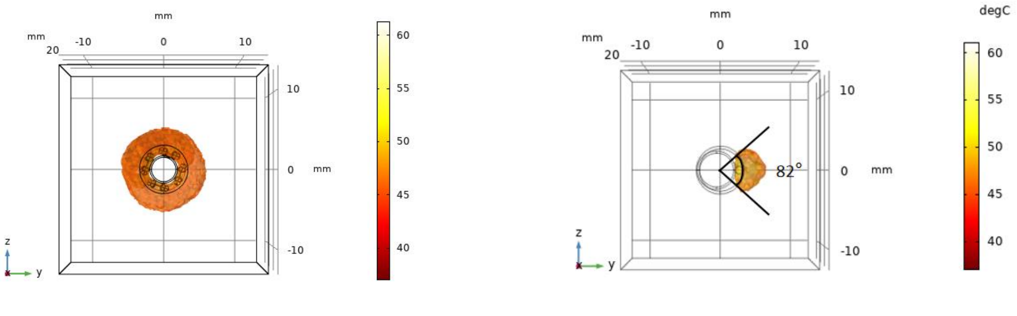


a. Netrod$^{TM}$ Six-electrode b. Single electrode

Figure 7 Comparison of the effective ablation angle between the Netrod$^{TM}$ Six-electrode and the single electrode

Comparing Figure.7(a) and (b), it can be found that the Netrod$^{TM}$ Six-electrode can work simultaneously, and the net basket configuration allows the electrodes to deliver RF energy close to the vessel wall. The Netrod$^{TM}$ Six-electrode ablation can achieve a comprehensive 360° ablation of sympathetic nerve, while the angle of single electrode of the same size is about 82°, which means that it takes about 5 times to achieve a complete 360° ablation of sympathetic nerve. The use of Netrod$^{TM}$ Six-electrode ablation overcomes the limitation of incomplete ablation of sympathetic nerve at one time.

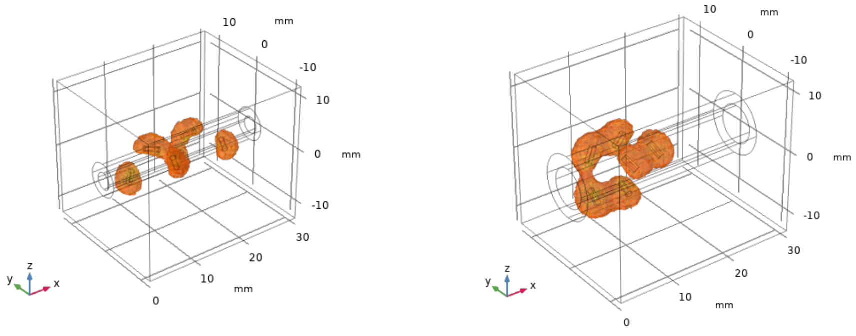

a. d=6mm b. d=10mm

Figure 8 Distribution of ablation areas of spiral six electrodes with lumen diameters of 6 and 10 mm

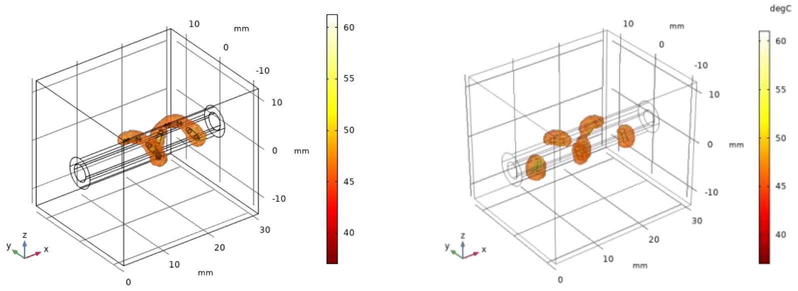


a. Netrod$^{TM}$ Six-electrode b. Spiral six electrodes

Figure 9 Comparison of the continuity of the ablation area between the Netrod$^{TM}$ Six-electrode and the spiral six electrodes

Comparing Figure.8(a) and (b), when the ablation time was 30 s, the ablation zone of the spiral six electrodes showed only partial continuity within the 6 mm diameter lumen, with gaps in the ablation zones of individual electrodes. This discontinuity arises because all electrodes in the spiral configuration are positioned on a single catheter, causing them to stretch laterally as the lumen diameter changes. In narrower lumens, the longitudinal positioning of the electrodes becomes dispersed, leading to scattered electrode positions and potential gaps in the ablation area. Figures 9(a) and 9(b) illustrate the continuity of the ablation zones between the Netrod™ Six-electrode and the spiral six electrodes at a renal artery diameter of 6 mm with an ablation time of 5 seconds. The ablation zone of the Netrod™ Six-electrode is completely continuous even within this short ablation time, while the spiral six electrodes fail to achieve continuous ablation. The Netrod™ Six-electrode, with each electrode positioned on individual net basket wires, can better conform to the vessel wall as the lumen changes, maintaining a consistent electrode arrangement. This results in superior continuity of the ablation area compared to the spiral electrodes.Unlike single and spiral configurations, the Netrod™ Six-electrode enables 360° ablation with a continuous ablation area.

### 3.3 Results of phantom experiments

As shown in Figure 10(a), during the ablation process, the six electrodes discharge to form a continuous 360° spiral ribbon, with the ablation ranges of the first and sixth electrodes overlapping radially. The RF ablation experiments yielded resulted in an ablation zone with a width of 6-8 mm and a depth of 4-6 mm, as depicted in Figure 10(b).

When comparing with the phantom experiment results, the width of the ablation zone was consistent, but the depth was significantly lower in the simulation. This discrepancy can be

attributed to the fact that phantom experiments allow for manual adjustments by the operator, enabling the Netrod™ Six-electrode to be positioned optimally against the vessel wall. In contrast, the simulation experiments exhibit slightly poorer wall apposition compared to the manually manipulated positions of the electrodes.

The results from the phantom experiments validate the continuous ablation zone of the Netrod™ Six-electrode observed in the simulation. These experiments demonstrate that, despite the discrepancies in depth, the width of the ablation zone remains consistent, further supporting the accuracy of the simulation model. By comparing the body model experiments with the simulation results, we can confirm the effectiveness of the six electrodes in achieving a 360° spiral ablation. This consistency not only enhances confidence in the simulation model but also provides a reliable basis for clinical applications, indicating that the electrodes can effectively control ablation range and depth during actual procedures.

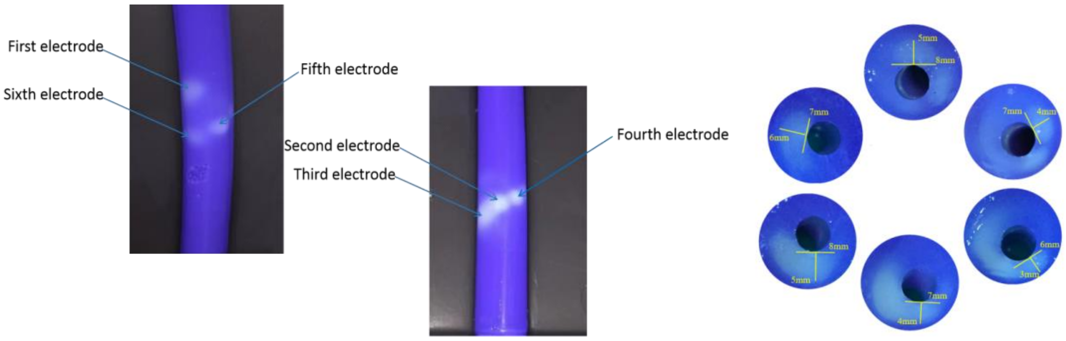


Figure 10 Ablation effect of body mold experiment

## 4 Discussion

This study compares the ablation characteristics of three RF applicators: the single electrode, spiral six electrodes, and the Netrod™ Six-electrode system. Results show that the Netrod™ Six-electrode system provides more comprehensive coverage and better continuity of the ablation zone compared to both the single electrode and the spiral six electrodes under the same setup. Lesion formation is influenced by several factors, including ablation power, ablation time, the number of electrodes, the area in contact with tissue, and blood flow.

AI Raisi et al. [28] reported a circumferential coverage angle of 54.90° ± 4.36° for a single-electrode RF application, encompassing approximately 15% of the arterial wall circumference. In contrast, the circumferential coverage angle achieved by the Netrod™ Six-electrode was 360°. Yanyan Cheng et al. noted that the placement of spiral electrodes could cause blood to flow spirally along the catheter, potentially resulting in more significant blood flow disturbances. Following balloon electrode placement in the renal artery, normal blood flow is obstructed, resulting in maximum blood flow velocity at the front of the electrode [29]. Additionally, since the four electrodes are not aligned in the same plane, a small amount of blood can still flow through. The simple structure of the single electrode results in less disruption of blood flow during the ablation process. Meanwhile, the mesh basket design of the Netrod™ Six-electrode effectively mitigates blood flow obstruction caused by both the spiral structure and the balloon.

However, our preliminary research has some limitations. First, we utilized a simplified model of the Netrod™ Six-electrode and the renal arteries, omitting the full structure of the Netrod™ Six-electrode and only simulating the electrode positions. Although the electrode positions can vary and adhere to the vessel wall according to the lumen diameter, the contact force between the electrodes and tissue was not considered in our model.

While simulation studies provide valuable insights, there remains a significant gap between these findings and their clinical application. In a clinical setting, factors such as patient-specific anatomy, variability in tissue properties, and real-time physiological responses can significantly influence the outcomes of RF ablation procedures. Additionally, the dynamic nature of blood flow and its interaction with the ablation device must be carefully assessed to optimize treatment effectiveness and minimize complications.

To bridge this gap, future studies should focus on integrating more complex and realistic models that account for these variables, including variations in blood flow dynamics and tissue response during ablation. Clinical trials will also be essential to validate the efficacy and safety of the Netrod™ Six-electrode in diverse patient populations demographics, ensuring that laboratory findings translate effectively into real-world applications.

## 5 Conclusion

Through mathematical modeling and phantom experiments, we established a novel renal denervation system based on the Netrod™ Six-electrode. This system utilizes automatic temperature control and a mesh basket structure to target the nerves around the renal arteries. Simulation studies of the Netrod™ Six-electrode demonstrated that heat distribution effectively achieved denervation of the renal sympathetic nerves while maintaining a constant electrode temperature of 60°C.

This paper emphasizes the feasibility of the proposed technique through mathematical modeling and phantom studies. Further clinical studies are needed imperative to validate the effectiveness and safety of the Netrod™ Six-electrode denervation system for blood pressure control management in clinical applications.

## References


[1] YANG X, LIN L, ZHANG Z, et al. Effects of catheter-based renal denervation on renin-aldosterone system, catecholamines, and electrolytes: A systematic review and meta-analysis [J]. J Clin Hypertens (Greenwich), 2022, 24(12): 1537-1546.

[2] SHARP A S P, TUNEV S, SCHLAICH M, et al. Histological evidence supporting the durability of successful radiofrequency renal denervation in a normotensive porcine model [J]. J Hypertens, 2022, 40(10): 2068-2075.

[3] DíAZ-MORALES N, BARANDA-ALONSO E M, MARTíNEZ-SALGADO C, et al. Renal sympathetic activity: A key modulator of pressure natriuresis in hypertension [J]. Biochem Pharmacol, 2023, 208: 115386.

[4] ESLER M D, KRUM H, SCHLAICH M, et al. Renal sympathetic denervation for treatment of drug-resistant hypertension: one-year results from the Symplicity HTN-2 randomized, controlled trial [J]. Circulation, 2012, 126(25): 2976-2982.

[5] ESLER M D, BöHM M, SIEVERT H, et al. Catheter-based renal denervation for treatment of patients with treatment-resistant hypertension: 36 month results from the SYMPLICITY HTN-2 randomized clinical trial [J]. Eur Heart J, 2014, 35(26): 1752-1759.

[6] KRUM H, SCHLAICH M P, SOBOTKA P A, et al. Percutaneous renal denervation in patients with treatment-resistant hypertension: final 3-year report of the Symplicity HTN-1 study [J]. Lancet, 2014, 383(9917): 622-629.

[7] LINZ D, HOHL M, ELLIOTT A D, et al. Modulation of renal sympathetic innervation: recent insights beyond blood pressure control [J]. Clin Auton Res, 2018, 28(4): 375-384.

[8] GöTZINGER F, KUNZ M, LAUDER L, et al. Radio frequency-based renal denervation: a story of simplicity? [J]. Future Cardiol, 2023, 19(9): 431-440.

[9] WHITE W B, GALIS Z S, HENEGAR J, et al. Renal denervation therapy for hypertension: pathways for moving development forward [J]. J Am Soc Hypertens, 2015, 9(5): 341-350.

[10] AZIZI M, SAPOVAL M, GOSSE P, et al. Optimum and stepped care standardised antihypertensive treatment with or without renal denervation for resistant hypertension (DENERHTN): a multicentre, open-label, randomised controlled trial [J]. Lancet, 2015, 385(9981): 1957-1965.

[11] KANDZARI D E, BöHM M, MAHFOUD F, et al. Effect of renal denervation on blood pressure in the presence of antihypertensive drugs: 6-month efficacy and safety results from the SPYRAL HTN-ON MED proof-of-concept randomised trial [J]. Lancet, 2018, 391(10137): 2346-2355.

[12] KARIO K, RUMOROSO J R, OKAWARA Y, et al. Renal Sympathetic Denervation in Patients with Resistant Hypertension: A Feasibility Study [J]. Pulse (Basel), 2019, 6(3-4): 137-143.

[13] WORTHLEY S G, TSIOUFIS C P, WORTHLEY M I, et al. Safety and efficacy of a multi-electrode renal sympathetic denervation system in resistant hypertension: the EnligHTN I trial [J]. Eur Heart J, 2013, 34(28): 2132-2140.

[14] Zhang J，Chen X，Guo ZF, et al. Development and experimental study of a new instrument used for percutaneous renal sympathetic radiofrequency ablation [J]. Intervent Radiol, 2016, 25(04): 341-344.

[15] FANG Z, MOSER M A J, ZHANG E, et al. Design of a Novel Electrode of Radiofrequency Ablation for Large Tumors: In Vitro Validation and Evaluation [J]. J Biomech Eng, 2019, 141(3).

[16] POP M, MOLCKOVSKY A, CHIN L, et al. Changes in dielectric properties at 460 kHz of kidney and fat during heating: importance for radio-frequency thermal therapy [J]. Phys Med Biol, 2003, 48(15): 2509-2525.

[17] GONZáLEZ-SUáREZ A, BERJANO E. Comparative Analysis of Different Methods of Modeling the Thermal Effect of Circulating Blood Flow During RF Cardiac Ablation [J]. IEEE Trans Biomed Eng, 2016, 63(2): 250-259.

[18] PLOURDE B D, VALLEZ L J, SUN B, et al. The Effect of Plaque Removal on Pressure Drop and Flow Rate through an Idealized Stenotic Lesion [J]. Biology and Medicine, 2015, 8(1).

[19] Dong T， Cheng YY， Tian Z, et al. Ex-vivo experimental study in hepatic vascular to simulate the radiofrequency ablation of renal sympathetic nerve [J]. Beijing Biomedical Engineering, 2018, 37(03): 265-272+319.

[20] DONG T, NAN Q, TIAN Z, et al. Radiofrequency ablation of renal sympathetic nerve: Numerical simulation and ex vivo experiments [J]. Advances in Mechanical Engineering, 2018, 10(6).

[21] SHAO Y L, LEO H L, CHUA K J. Studying the thermal performance of a bipolar radiofrequency ablation with an improved electrode matrix system: In vitro experiments and modelling [J]. Applied Thermal Engineering, 2017, 116.

[22] ZHENG F, BING Z, MICHAEL M, et al. Design of a Novel Electrode of Radiofrequency Ablation for Large Tumors: A Finite Element Study [J]. Journal of Engineering and Science in Medical Diagnostics and Therapy, 2018, 1(1).

[23] NEGUSSIE A H, PARTANEN A, MIKHAIL A S, et al. Thermochromic tissue-mimicking phantom for optimisation of thermal tumour ablation [J]. Int J Hyperthermia, 2016, 32(3): 239-243.

[24] CHEN Y, YOUN P, FURGESON D Y. Thermo-targeted drug delivery of geldanamycin to hyperthermic tumor margins with diblock elastin-based biopolymers [J]. J Control Release, 2011, 155(2): 175-183.

[25] Huang Y, Hu B, Shen E. Experimental study of interaction between radiofrequency ablation and realtime temperature monitoring. Chinese Journal of Interventional Imaging and Therapy 2010;7(03):296-300.

[26] Zhang BL, Hu B, Kuang SL,Huang Y,Wu R,Jiang LX,et al. A tissue-mimicking phantom for visualizing coagulation temperature distribution at the same range of biological tissue during thermal ablation. Chinese Journal of Medical Imaging Technology 2007;(09):1405-1408.

[27] CHENG Y, LIU H, TIAN Z, et al. Comparison of ablation characteristics of three different radiofrequency applicators in renal sympathetic denervation [J]. Int J Hyperthermia, 2021, 38(1): 1251-1262.

[28] AL RAISI S I, POULIOPOULOS J, BARRY M T, et al. Renal Artery Branch Denervation: Evaluation of Lesion Characteristics Using a Thermochromic Liquid Crystal Phantom Model [J]. Heart Lung Circ, 2020, 29(3): 445-451.

[29] CHENG Y, LIU H, TIAN Z, et al. Evaluating the thermal performance of a balloon-based renal sympathetic denervation system with array electrodes: a finite element study [J]. Electromagn Biol Med, 2021, 40(4): 488-501.

[30] BAIK J, SONG W H, YIM D, et al. Laparoscopic Renal Denervation System for Treating Resistant Hypertension: Overcoming Limitations of Catheter-Based Approaches [J]. IEEE Trans Biomed Eng, 2020, 67(12): 3425-3437.

Manuscript Click here to access/download;Manuscript;Revised manuscript.docx

# Study on the efficacy of Netrod$^{TM}$ Six-electrode Radiofrequency Renal Denervation system on renal artery vessels

Xiaohua Song[ac1] Jiulin Guo[b1] Hongguang Cao[b] Qun Nan[a*1]

[a] College of Chemistry and Life Science, Beijing University of Technology, Beijing, China

[b] Shanghai Golden Leaf MedTech CO., Ltd, Shanghai, China

[c] Industrial Informatization and Science and Technology Bureau of Changqing District, Jinan, China

**Abstract:** The principle of NetrodTM Renal Denervation RF Generator is based on the heating of radiofrequency electrodes to achieve the therapeutic temperature for peripheral nerve ablation with minimal impact on intra-arterial blood flow. The extravascular heat distribution and continuous ablation were evaluated using COMSOL Multiphysics with an idealized 3D model and validated in ex vivo experiment. The safety of ablation was verified by H&E staining of hypertensive dog model in treatment group of animal experiments, and changes in blood pressure before and after the procedure were used to characterize the efficacy. The simulation results showed that the maximum injury depth range of the Netrod$^{TM}$ Six-electrode is 5.08mm and the depth of the ablation zone obtained in the Phantom experiment was 3-6mm. Circumferential coverage was up to 91.99% and the ablation zone was continuously. In animal experiments, eight animals in the treatment group had significantly lower blood pressure (88.9%). Nerve cells in nerve bundles show vacuolar changes and thickening of peripheral sheaths after peripheral nerve ablation of renal arteries.The NetrodTM Six-electrode presented here has potential to effectively and safely ablate renal nerves. Systematic ablation can reach the target temperature in a shorter period of time, with faster warming, greater peripheral coverage of the lesion, and a more continuous ablation zone.

**Keywords:** Radio Frequency Ablation; Renal Denervation; Resistant hypertension; Temperature controlled; Netrod$^{TM}$ Six-electrode.

# Introduction

Hypertension is a cardiovascular syndrome with elevated arterial pressure in the body circulation as the main clinical manifestation and is an important risk factor for cardiovascular and cerebrovascular diseases. Hypertension is a common chronic disease that continues to increase in prevalence worldwide and is expected to reach 1.56 billion people by 2025, with refractory hypertension(RH) being particularly challenging to treat.[1]. Refractory hypertension(RH) is defined as hypertension in which blood pressure cannot be achieved even after adequate and reasonable combination of at least 3 antihypertensive drugs (including diuretics) based on dietary modification and lifestyle improvement[2]. RH accounts for about 5-15% of hypertensive patients, and some sources report that it can reach up to 20%-30%. There is still a clinical need for new easy, safe and effective treatments for hypertension.

Excessive sympathetic activation plays a significant role in the development and maintenance of hypertension [3-5]. Several studies have indicated that blocking sympathetic nerves may serve as a viable target for hypertension treatment [6-8]. Renal denervation (RDN) is a minimally invasive procedure that reduces blood pressure by modulating sympathetic hyperexcitability [9]. This technique is gaining acceptance and widespread application in clinical settings across various

* Corresponding author College of Chemistry and Life Science, Beijing University of Technology, Beijing, 100124, China

E-mail addresses: nanqun@ bjut. edu. cn

[1] Xiaohua Song and Jiulin Guo are Co-first authors

countries [10].Other studies[11] have also shown that RDN has the potential to extend to other autonomic nervous system disorders such as atrial fibrillation[12,13], heart failure[14,15] and tachycardia[16].

Although research related to RDN is still in its early stages, several new devices have been developed in recent years [17]. Common radiofrequency (RF) catheter ablation devices include the Symplicity System, Symplicity Spyral System, EnligHTN System, FlashPoint™ System, and Iberis System. We have developed the Netrod™ Six-electrode Radiofrequency Renal Denervation system and have investigated the safety and efficacy of the system in simulations, phantom experiment, and animal studies.

In the following sections, we will detail the numerical simulations and validation experiments conducted with the Netrod™ Six-electrode. This research aims to provide a scientific basis for assessing the clinical efficacy of renal sympathetic nerve ablation using the Netrod™ Six-electrode.

# Methods

## Equipment Introduction

Netrod™ Six-electrode Radiofrequency Renal Denervation system (Shanghai Golden Leaf MedTech Co. Ltd) includes Netrod™ Renal Denervation RF Generator and Netrod™ Six-electrode Radiofrequency Renal Denervation catheter (Fig 1).

[Here Fig. 1]

**Figure 1 Radiofrequency ablation mainframe and Netrod™ Six-electrode structure diagram**

The generator primarily consists of a microcontroller, a signal source circuit, a power drive circuit, a power output circuit, a detection circuit, and a display operation control unit. The detection circuit mainly detects monitors voltage, current, and temperature levels (Fig 2).

[Here Fig. 2]

**Figure 2 Netrod™ Renal Denervation RF Generator components**

The microcontroller is the core component of the generator. It not only processes the voltage, current, and temperature data transmitted by the detection circuit, but also outputs impedance, temperature, and power data to the display operation control unit. Furthermore, it intelligently adjusts the power output of the signal sources based on temperature and impedance to maintain the electrode surface temperature at a constant value, thereby ensuring safety during operation. The display operation control unit real-time records and displays the temperature, impedance, and power of each electrode during the radiofrequency ablation, and draws relevant curves (Fig 3), allowing medical staff to monitor the working status of the system in real-time through the display.

[Here Fig. 3]

**Figure 3 120 seconds of radiofrequency ablation, the power(P), temperature(T), and impedance(Z) of electrode 4 change over time**

The radiofrequency ablation catheter is composed of a central wire drawing with a developing head, a mesh tube connector, an electrode, a mesh basket bracket, a catheter sleeve, a seven-hole tube, and a handle assembly (Fig 4).

[Here Fig. 4]

**Figure 4 Netrod™ Six-electrode Basket Radiofrequency Renal Denervation Catheter. 1: Central wire drawing with a developing head; 2: Mesh basket bracket; 3: Electrode; 4: Seven-hole tube; 5: Adjustment knob; 6: Handle**

[Here Fig. 5]

**Figure 5 Basket catheter ablation of renal sympathetic nerves. A. Renal sympathetic nerve distribution; B,C. The basket catheter enters the branch for ablation; D. The basket**

**catheter expands the basket stent to perform ablation in the main trunk of the renal artery.**

# Finite element method modeling

We use COMSOL Multiphysics software (COMSOL, Burlington MA, USA), a finite element simulation software, to perform electromagnetic field and temperature field coupling calculations.

In radiofrequency ablation therapy, thermal energy is generated by converting the electrical energy that is applied as the application of radiofrequency current to the target renal artery tissue. The electric field control equation[18] can be calculated by solving the following Laplace equation. The frequency of the RF current is set to 470 KHz[19] , and the biological medium can be considered resistive because the displacement current is much smaller than the conduction current and thus can be neglected[20]. The distributed heat source Qr is given by Eq. (1)

$$Q_r = J \cdot E \tag{1}$$

Where J is the current density, measured in A/m$^2$; Qr is the distributed heat source, measured in J. The voltage is obtained from the solved Laplace equation, which is the controlling equation for solving the electric field problem in the presence of the internal power supply; see Eq. (2) and (3).

$$\nabla \cdot (\sigma \nabla V) = 0 \tag{2}$$

$$E = -\nabla V \tag{3}$$

Where V is the voltage, measured in V; E is the electric field strength, measured in V/m;$\sigma$ is the electrical conductivity, measured in S/m。 In the model, the boundary conditions at the current interface are set as follows:

$$\begin{array}{ll} V = 0 & \text{All external surfaces of the abdominal cavity} \\ V = V_0 & \text{Six electrodes outer surface} \\ \mathbf{n} \cdot J=0 & \text{All other domain boundaries} \end{array} \tag{4}$$

The initial potential is set to 0 V, the outer surface of the electrodes is set to a voltage value with temperature control. In the entire model, all the outer surfaces of the tissue are grounded with zero current density.

In the simulation, an idealized three-dimensional vascular geometry model of the renal artery was constructed, as shown in Fig 6. The tissue included two major parts, the renal artery（6mm diameter) and the abdominal cavity (26mm×26mm×30mm), which was constructed as the external environment for the simulation. The arterial inlet flow rate was set to 0.5 m/s, and the ablation time was set to 120s.

[Here Fig. 6]

**Figure 6 Three-dimensional renal artery tissue and net basket-based RF electrode model**

**Table 1 shows material property parameters of the abdominal part, blood and electrodes**[21-25].

| Material Properties | ρ(kg/m$^3$) | σ (S/m) | C(J/kg·°C) | K(W/(m·°C) | $\xi_r$ |
|---|---|---|---|---|---|
| Blood | 1050 | 0.99 | 4180 | 0.54 | 58.3 |
| Abdominal cavity | 1060 | 1.69 | 3600 | 0.512 | 43.03 |
| Agar | 1300 | 2.3 | 4182 | 0.6 | 57.99 |
| Gold Electrode | 19300 | 4.1e7 | 127 | 315 | 1 |

Where ρ is the density, Kg/m$^3$; σ is the conductivity, S/m; C is the specific heat capacity, J/kg·°C; K is the thermal conductivity, m·°C; $\xi_r$ is the relative dielectric constant.

# Phantom experiment validation

Tissue-mimicking phantoms play a crucial role in the development, performance testing, quality assurance, and formulation of effective treatment plans for RF ablation devices[26]. Given that the walls of real human renal vessels are very thin, the wall thickness is often neglected in experimental models. In this study, we utilized a cylindrical mold with a vascular channel, where the surrounding area simulates human abdominal tissue. The entrance diameter of the vascular channel is 6 mm, corresponding to the dimensions of the simulation model. Polyacrylamide is particularly advantageous due to its stability at high temperatures. Many researchers have reported successful applications of polyacrylamide-based transparent tissue-mimicking molds, incorporating protein as a temperature indicator for thermal ablation studies[27]. The raw materials and formulation details for the specific mold used in this study are presented in Table 2.[28]

**Table 2 Proportioning table for the preparation of 100 ml solution**[29]

| Substance Name | Weighing |
|---|---|
| Citric acid | 2.49g |
| Sodium citrat | 2.66g |
| Acrylamide | 7.62g |
| Methylene-bisacrylamide | 0.4g |
| Bovine serum protein | 2.0g |
| Degassed water | 100ml |
| Ascorbic acid | 0.1g |
| 1%$FeSO_4$ | 1.0ml |
| 3%$H_2O_2$ | 1.0ml |

The target temperature for the electrode was set at 60˚C, and the experiment was conducted for a duration of 120 seconds. For thermal damage analysis, we evaluated the depth of thermal damage in the body mold through post-experimental assessment. The body mold changes color from blue to white when the temperature exceeds 45°C. Therefore, the color change on the surface of the body mold served as an indicator of the ablation effect.

# Animal study

Dogs subjected to animal testing and experimental protocols were treated in strict accordance approved by the Institutional Animal Care Committee of Shanghai Harborside Medical Technology Co., Ltd., based on the Institutional Animal Care and Use Committees（IACUC）Animal Care and Use Program on Animal Experimentation (approval No.IACUC-2021-003).

A total of 18 female experimental dogs (treatment group 101-109 and control group 201-209, 9 animals each) that had successfully established hypertension models were included in this experiment divided into three points of time, with 6 dogs in each point of time (3 animals each in the treatment group and the control group). The three points of time were 7 days (101-103 for the treatment group and 201-203 for the control group), 30 days (104-106 for the treatment group and 204-206 for the control group) and 60 days (107-109 for the treatment group and 207-209 for the control group). Under X-ray fluoroscopy, the left and right renal arteries of the animals in the treatment group were ablated with the system, and the operational performance of the test article was evaluated, and the Digital Subtraction Angiography(DSA) images are shown in Fig 7(a)-(d).

The duration of ablation was set to 120s. As a measure of efficacy, blood pressure was measured at regular intervals before and during the post-test observation period, and we measured the change in blood pressure at three points of time after RDN. During survival, animals were observed clinically. To investigate the short- term, medium- term, and long-term safety of the test article, three points of time, 7, 30, and 60 days, were included in this experiment. On postoperative days 7 (±1), 30 (±3) and 60 (±5) (with the day of surgery as 0 day), renal arteriograms were performed in animals scheduled for dissection to visualize the test site and renal arteries with nerves were harvested for immuno-histochemical evaluation. Animals were euthanized by deep anesthesia and an overdose of potassium chloride for autopsy and histopathological analysis. Quantification of protein denaturation and necrosis of renal sympathetic nerves around renal arteries were using hematoxylin-eosin (H&E) staining.

[Here Fig.7]

**Figure 7 Typical images of preanatomic renal arteriogram**

# Results

## Simulation results of Netrod™ Six-electrode

As the ablation time increases, the ablation area expands, with data detailed in Table 3.

**Table 3 Ablation of electrodes at different times**

| | Electrode temperature/˚C | Distance between 45˚C boundary and electrodes/mm |
|---|---|---|
| 5s | 48.6 | 0.23 |
| 30s | 60.0 | 2.69 |
| 60s | 60.0 | 3.78 |
| 120s | 60.0 | 5.08 |

With prolonged ablation time, the boundary of the region where the temperature reaches 45°C expands. Since temperature-controlled RF ablation was employed, the maximum temperature was confined to the vicinity of the electrodes, as shown in Table 3. The Netrod™ Six-electrode device can reach temperatures up to 45°C at distances of approximately 5.08 mm (>4 mm) from the electrode.

As shown in Fig 8， The Netrod™ Six-electrode can work simultaneously, and the mesh basket structure allows the electrodes to deliver RF energy close to the vessel wall.Netrod™ Six-electrodecan achieve 360° total ablation of sympathetic nerves in 30s.

[Here Fig.8]

**Figure 8 The effective ablation angle of Netrod™ Six-electrode(t=30s)**

The continuity of the Netrod™ Six-electrode ablation zone at an ablation time of 30s is shown in Fig 9. Each of the Netrod™ Six-electrode sits on a separate mesh basket of wires, which better conforms to the vessel wall as the lumen changes, keeping the electrode arrangement consistent.

[Here Fig.9]

**Figure 9 The continuity of the ablation area between the Netrod™ Six-electrode(t=30s)**

## Results of phantom experiments

As shown in Fig 10, during the ablation process, the six electrodes discharge to form a

continuous 360° spiral ribbon, with the ablation ranges of the first and sixth electrodes overlapping radially. The RF ablation experiments yielded resulted in an ablation zone with a width of 6-8 mm and a depth of 3-6 mm, as depicted in Fig 10.

The results from the phantom experiments validate the continuous ablation zone of the Netrod™ Six-electrode observed in the simulation. These experiments demonstrate that, despite the discrepancies in depth, the width of the ablation zone remains consistent, further supporting the accuracy of the simulation model. By comparing the body model experiments with the simulation results, we can confirm the effectiveness of the six electrodes in achieving a 360° spiral ablation. This consistency not only enhances confidence in the simulation model but also provides a reliable basis for clinical applications, indicating that the electrodes can effectively control ablation range and depth during actual procedures.

[Here Fig.10]

**Figure 10 Ablation effect of body mold experiment**

# Animal experiment results

The surgeon performed radiofrequency ablation of the left and right renal arteries of the hypertensive model animals according to the protocol (9 animals in the treatment group) or a sham procedure with renal artery angiography only (9 animals in the control group). There were no intraoperative abnormalities or serious adverse events related to animal vital signs, and the operability of the system was rated as excellent by the surgeon.

The group of the 18 experimental animals was mentioned in the previous section. The blood pressure of the animals was measured before and periodically after the procedure, and the blood pressure reduction data at different points of time are shown in Fig 11. Of the three animals in the test group at the point of time of 7 (±1) days, pre-anatomic diastolic and mean arterial pressures in animals 101 may not be statistically different relative to pre-ablation due to individual differences ($P>0.05$). In the other 2 animals, systolic, diastolic, and mean arterial pressure were significantly lower than before ablation ($P<0.05$). Blood pressure was significantly lower than before ablation ($P<0.05$) in all 6 animals in the test group at the 30 (±3) and 60 (±5) day point of time. Pre-ablation systolic, diastolic, and mean arterial pressures in the nine control animals during this procedure were only marginally reduced or increased relative to pre-sham procedure, with no significant differences ($P>0.05$).

[Here Fig.11]

**Figure 11 Values of blood pressure reduction in animals of the treatment group at different point of time**

The body weights of the experimental animals were not significantly abnormal during the experimental period, and the clinicopathological indices of the animals did not show any significant health-related abnormalities in the preoperative and pre-dissection test results. During the postoperative observation period, the experimental animals did not show any abnormalities related to the treatment. No abnormalities such as stenosis or dissection were observed in the renal arteries of the animals when the angiography was performed before dissection, and no abnormalities were observed when the animals were dissected for observation and sampling. The above experiments prove the safety of the device. The results of the validity analysis in the histopathological analysis showed that nerve fiber damage around bilateral renal artery vessels in animals of the treatment group at each point of time, showing different degrees of vacuolar degeneration, hyaline degeneration and coagulative necrosis. Degeneration of nerve fibers around the renal artery vessels was also seen in pathological section samples from most of the control animals, which was considered to be nerve injury reaction caused by the sham operation itself. In the histopathological

analysis, the data we measured to obtain the maximum distance from the ablation point to the nerve fiber damage in the treatment group are shown in Fig 12.

[Here Fig.12]

**Figure 12 Distance of nerve damage at different points of time**

The distance of nerve injury at the three points of time was not particularly different, and it was inferred that reversible repair of the injured nerve would not occur over time. The results of H&E staining of the ablation sites showed that the intima of the renal artery vessels in all groups of animals showed varying degrees of thickening and inflammatory infiltration. None of them showed desquamation, dissection, calcification or organization. Occasional there is thrombus formation in the lumen, which was considered to be caused by blood clotting in the blood vessels during animal dissection. Vascular injury and inflammatory infiltration of the tunica media were present to varying degrees in all groups of animals and no contractures or pseudoaneurysm were present. Occasional hyaline degeneration of the muscularis was seen in both groups, which was considered to be caused by ablation or the procedure itself. Different degrees of Necrosis of perivascular tissue, inflammatory infiltration, fibrosis and fatty infiltration were seen in the adventitia and perivascular tissue of renal arteries in both groups, and the overall pathological irritant response was more pronounced in the test group than in the blank control group. An overview of the efficacy and safety index pathological results is shown in Fig 13 for representative samples at different time periods, with animal number 101 (7 days), 104 (30 days) and 107 (60 days) for the treatment group and 201 (7 days), 204 (30 days) and 207 (60 days) for the blank control group.

[Here Fig.13]

**Figure 1 H&E staining of nerves (inflammatory cells shown by yellow arrows; tissue necrosis shown by blue arrows; fatty infiltration shown by green arrows; intimal thickening shown by red arrows)**

Figure 14A and B show the peripheral nerve of the renal artery after ablation, and a large number of vacuolar changes of nerve cells in the nerve bundle are seen; C and D are the peripheral nerve of the renal artery in the blank control group, and no significant abnormal changes were seen in the nerve bundles. The H&E staining pathological pictures of the canine renal artery peripheral nerve 42 days after the controlled experimental radiofrequency ablation of the renal artery are shown in Figure 15. The peripheral nerve bundle of the renal artery in the test group showed morphological changes and thickening of the peripheral sheath. No significant abnormal changes were seen in the peripheral nerve bundle of the renal artery in the blank control group. Combining the pathological results of the above efficacy and safety indicators, it was inferred that the treatment could effectively ablate perivascular nerve tissue and cause irreversible damage to nerve fibers. The degree of local inflammation and other pathological reactions in the treatment group was significantly greater than that in the blank control group at all points of time, which was considered to be caused by intraoperative ablation damage to tissues, but did not show significant safety problems, and all of them decreased with the prolongation of postoperative time.

[Here Fig.14]

**Figure 2 H&E staining of the peripheral nerve of the renal artery**

[Here Fig.15]

**Figure 3 H&E staining of the peripheral nerve of the renal artery after 42 days**

# Discussion

AI Raisi et al. [30] reported a circumferential coverage angle of 54.90° ± 4.36° for a single-electrode RF application, encompassing approximately 15% of the arterial wall circumference. In contrast, the circumferential coverage angle achieved by the Netrod™ Six-electrode was 360°. Yanyan Cheng et al. noted that the placement of spiral electrodes could cause blood to flow spirally

along the catheter, potentially resulting in more significant blood flow disturbances. Following balloon electrode placement in the renal artery, normal blood flow is obstructed, resulting in maximum blood flow velocity at the front of the electrode [31]. The mesh basket design of the Netrod™ Six-electrode effectively mitigates blood flow obstruction caused by both the spiral structure and the balloon.

The relevant parameters (including size and material) of the electrodes used in the ex vivo and animal experiments are the same as those of the electrodes used in the simulation. However, our preliminary research has some limitations. First, we utilized a simplified model of the Netrod™ Six-electrode and the renal arteries, omitting the full structure of the Netrod™ Six-electrode and only simulating the electrode positions. Although the electrode positions can vary and adhere to the vessel wall according to the lumen diameter, the contact force between the electrodes and tissue was not considered in our model. While simulation studies provide valuable insights, there remains a significant gap between these findings and their clinical application. In a clinical setting, factors such as patient-specific anatomy, variability in tissue properties, and real-time physiological responses can significantly influence the outcomes of RF ablation procedures. Additionally, the dynamic nature of blood flow and its interaction with the ablation device must be carefully assessed to optimize treatment effectiveness and minimize complications. Finally，the animal experiment were selected from 18 female dogs, and it was not possible to determine whether gender had an effect on the results of the experiment. And experimental dogs may not reflect the characteristics of human arteries with hypertension. Depth data on the canine model does not fully reflect ablation depths in other animals and in humans.

To bridge this gap, future studies should focus on integrating more complex and realistic models that account for these variables, including variations in blood flow dynamics and tissue response during ablation. Clinical trials will also be essential to validate the efficacy and safety of the Netrod™ Six-electrode in diverse patient populations demographics, ensuring that laboratory findings translate effectively into real-world applications.

# Conclusion

Through mathematical modeling, phantom experiments and animal study, we established a novel renal denervation system based on the Netrod™ Six-electrode. This system utilizes automatic temperature control and a mesh basket structure to target the nerves around the renal arteries. Simulation studies of the Netrod™ Six-electrode demonstrated that heat distribution effectively achieved denervation of the renal sympathetic nerves while maintaining a constant electrode temperature of 60°C.

This paper focuses on mathematical modeling, phantom experiments and animal studies to demonstrate the feasibility of the proposed new technique. We performed renal denervation in hypertensive dogs model and in an open surgical setting to show the efficacy and safety of the system in vivo. Further clinical studies are needed to validate the effectiveness and safety of the NetrodTM Six-electrode denervation system for blood pressure control in vivo.

# Data Availability Statement

All relevant data are within the paper.

# Competing interests

The authors have declared that no competing interests exist.

# Funding:

This work was supported by the National Natural Science Foundation of China [31771021,

11832003].The funder had role in study design, data collection and analysis.

# Author Contributions

Conceptualization: Xiaohua Song, Jiulin Guo, Hongguang Cao, Qun Nan

Data curation: Xiaohua Song, Jiulin Guo, Hongguang Cao, Qun Nan

Formal analysis: Xiaohua Song, Qun Nan

Writing-original draft: Xiaohua Song

Writing- review & editing: Xiaohua Song, Jiulin Guo, Hongguang Cao, Qun Nan

# References

[1] KEARNEY P M, WHELTON M, REYNOLDS K, et al. Global burden of hypertension: analysis of worldwide data [J]. Lancet, 2005, 365(9455): 217-23.

[2] CALHOUN D A, JONES D, TEXTOR S, et al. Resistant hypertension: diagnosis, evaluation, and treatment: a scientific statement from the American Heart Association Professional Education Committee of the Council for High Blood Pressure Research [J]. Circulation, 2008, 117(25): e510-26.

[3] YANG X, LIN L, ZHANG Z, et al. Effects of catheter-based renal denervation on renin-aldosterone system, catecholamines, and electrolytes: A systematic review and meta-analysis [J]. J Clin Hypertens (Greenwich), 2022, 24(12): 1537-1546.

[4] SHARP A S P, TUNEV S, SCHLAICH M, et al. Histological evidence supporting the durability of successful radiofrequency renal denervation in a normotensive porcine model [J]. J Hypertens, 2022, 40(10): 2068-2075.

[5] DíAZ-MORALES N, BARANDA-ALONSO E M, MARTíNEZ-SALGADO C, et al. Renal sympathetic activity: A key modulator of pressure natriuresis in hypertension [J]. Biochem Pharmacol, 2023, 208: 115386.

[6] ESLER M D, KRUM H, SCHLAICH M, et al. Renal sympathetic denervation for treatment of drug-resistant hypertension: one-year results from the Symplicity HTN-2 randomized, controlled trial [J]. Circulation, 2012, 126(25): 2976-2982.

[7] ESLER M D, BöHM M, SIEVERT H, et al. Catheter-based renal denervation for treatment of patients with treatment-resistant hypertension: 36 month results from the SYMPLICITY HTN-2 randomized clinical trial [J]. Eur Heart J, 2014, 35(26): 1752-1759.

[8] KRUM H, SCHLAICH M P, SOBOTKA P A, et al. Percutaneous renal denervation in patients with treatment-resistant hypertension: final 3-year report of the Symplicity HTN-1 study [J]. Lancet, 2014, 383(9917): 622-629.

[9] LINZ D, HOHL M, ELLIOTT A D, et al. Modulation of renal sympathetic innervation: recent insights beyond blood pressure control [J]. Clin Auton Res, 2018, 28(4): 375-384.

[10] GöTZINGER F, KUNZ M, LAUDER L, et al. Radio frequency-based renal denervation: a story of simplicity? [J]. Future Cardiol, 2023, 19(9): 431-440.

[11] GOYAL A, JAIN H, VERMA A, et al. The role of renal denervation in cardiology and beyond: An updated comprehensive review and future directives. Curr Probl Cardiol.2024 Feb;49(2):102196.

[12] ZUHAIR M ,KEENE D ,KANAGARATNAM P , et al.Percutaneous Neuromodulation for Atrial Fibrillation.[J].Cardiac electrophysiology clinics,2024,16(3):281-296.

[13] DOIRON E J ,LI Z ,YU X , et al.Early Renal Denervation Attenuates Cardiac Dysfunction in Heart Failure With Preserved Ejection Fraction.[J].Journal of the American Heart Association,2024,13(4):e032646-e032646.

[14] YING W ,MEIYAN S ,MEIFANG W , et al.Advances in device-based treatment of heart failure with preserved ejection fraction: evidence from clinical trials.[J].ESC heart failure,2023,11(1):13-27.

[15] LIANG X, SHANG S, BAI Z, et al. Effects of Renal Denervation on Gap Junction in High-Pacing-Induced Heart Failure Dogs. Anatol J Cardiol. 2024 Jul 16;28(9):429–36.

[16] TAKAHASHI K ,EGAMI Y ,NISHINO M , et al.Clinical impact of stellate ganglion phototherapy on ventricular tachycardia storm requiring mechanical circulatory support devices: a case report.[J].European heart journal. Case reports,2024,8(4):ytae177-ytae177.

[17] WHITE W B, GALIS Z S, HENEGAR J, et al. Renal denervation therapy for hypertension: pathways for moving development forward [J]. J Am Soc Hypertens, 2015, 9(5): 341-350.

[18] FANG Z, MOSER M A J, ZHANG E, et al. Design of a Novel Electrode of Radiofrequency Ablation for Large Tumors: In Vitro Validation and Evaluation [J]. J Biomech Eng, 2019, 141(3).

[19] POP M, MOLCKOVSKY A, CHIN L, et al. Changes in dielectric properties at 460 kHz of kidney and fat during heating: importance for radio-frequency thermal therapy [J]. Phys Med Biol, 2003, 48(15): 2509-2525.

[20] GONZáLEZ-SUáREZ A, BERJANO E. Comparative Analysis of Different Methods of Modeling the Thermal Effect of Circulating Blood Flow During RF Cardiac Ablation [J]. IEEE Trans Biomed Eng, 2016, 63(2): 250-259.

[21] PLOURDE B D, VALLEZ L J, SUN B, et al. The Effect of Plaque Removal on Pressure Drop and Flow Rate through an Idealized Stenotic Lesion [J]. Biology and Medicine, 2015, 8(1).

[22] Dong T， Cheng YY， Tian Z, et al. Ex-vivo experimental study in hepatic vascular to simulate the radiofrequency ablation of renal sympathetic nerve [J]. Beijing Biomedical Engineering, 2018, 37(03): 265-272+319.

[23] DONG T, NAN Q, TIAN Z, et al. Radiofrequency ablation of renal sympathetic nerve: Numerical simulation and ex vivo experiments [J]. Advances in Mechanical Engineering, 2018, 10(6).

[24] SHAO Y L, LEO H L, CHUA K J. Studying the thermal performance of a bipolar radiofrequency ablation with an improved electrode matrix system: In vitro experiments and modelling [J]. Applied Thermal Engineering, 2017, 116.

[25] ZHENG F, BING Z, MICHAEL M, et al. Design of a Novel Electrode of Radiofrequency Ablation for Large Tumors: A Finite Element Study [J]. Journal of Engineering and Science in Medical Diagnostics and Therapy, 2018, 1(1).

[26] NEGUSSIE A H, PARTANEN A, MIKHAIL A S, et al. Thermochromic tissue-mimicking phantom for optimisation of thermal tumour ablation [J]. Int J Hyperthermia, 2016, 32(3): 239-243.

[27] CHEN Y, YOUN P, FURGESON D Y. Thermo-targeted drug delivery of geldanamycin to hyperthermic tumor margins with diblock elastin-based biopolymers [J]. J Control Release, 2011, 155(2): 175-183.

[28] Huang Y, Hu B, Shen E. Experimental study of interaction between radiofrequency ablation and realtime temperature monitoring. Chinese Journal of Interventional Imaging and Therapy 2010;7(03):296-300.

[29] Zhang BL, Hu B, Kuang SL,Huang Y,Wu R,Jiang LX,et al. A tissue-mimicking phantom for visualizing coagulation temperature distribution at the same range of biological tissue during thermal ablation. Chinese Journal of Medical Imaging Technology 2007;(09):1405-1408.

[30] AL RAISI S I, POULIOPOULOS J, BARRY M T, et al. Renal Artery Branch Denervation: Evaluation of Lesion Characteristics Using a Thermochromic Liquid Crystal Phantom Model [J]. Heart Lung Circ, 2020, 29(3): 445-451.

[31] CHENG Y, LIU H, TIAN Z, et al. Evaluating the thermal performance of a balloon-based renal sympathetic denervation system with array electrodes: a finite element study [J]. Electromagn Biol Med, 2021, 40(4): 488-501.

Figure Click here to access/download;Figure;S1_raw_images.pdf

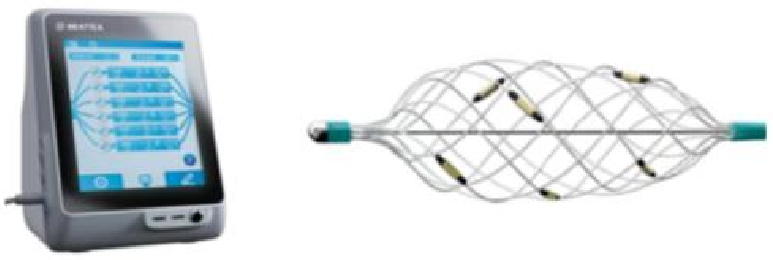

**Figure 1**

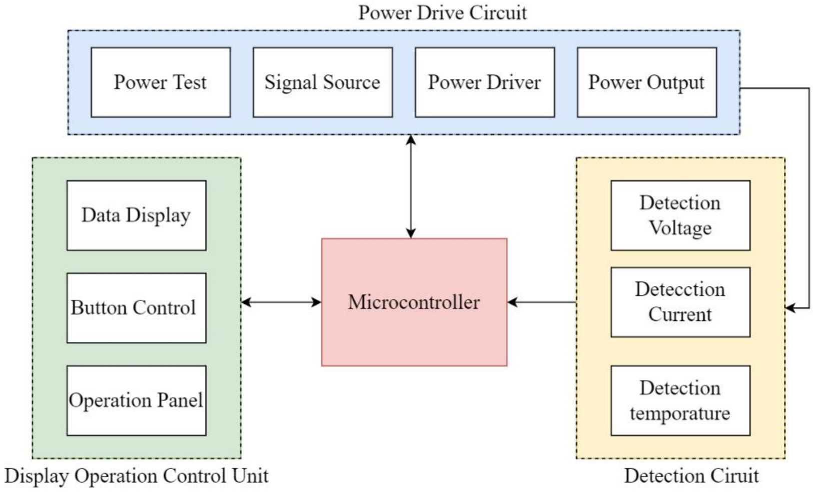


**Figure 2**

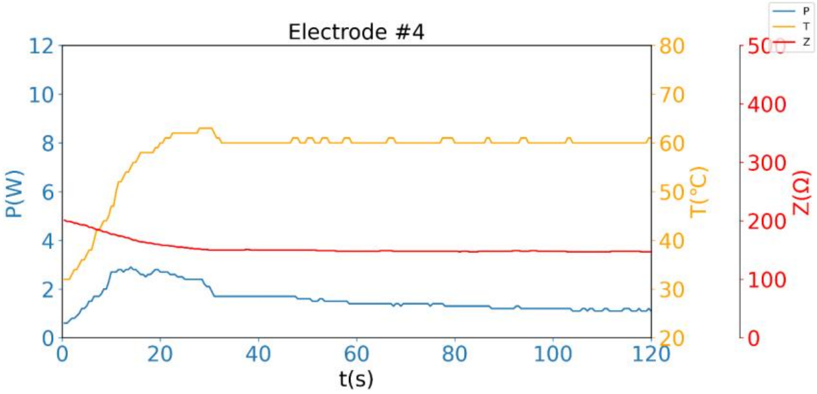


**Figure 3**

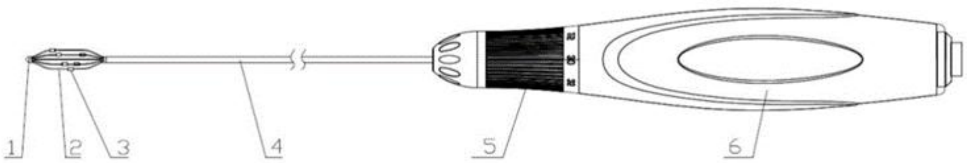


**Figure 4**

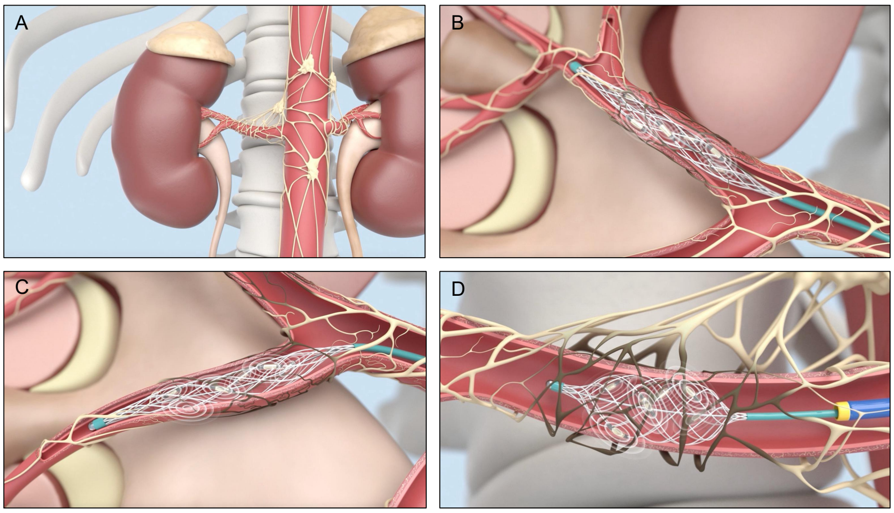


**Figure 5**

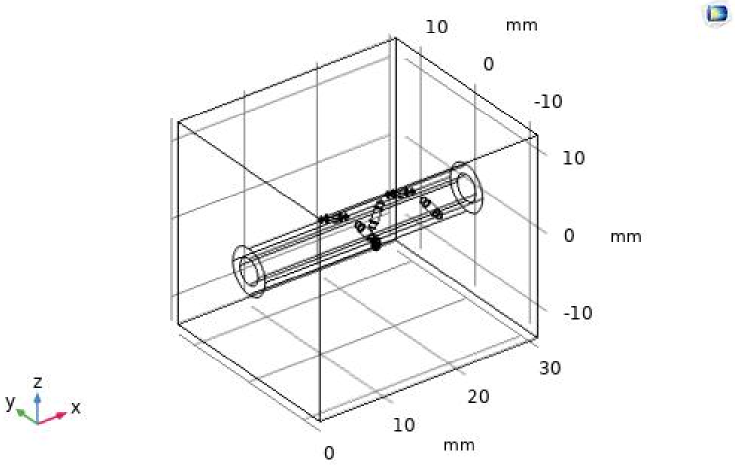


**Figure 6**

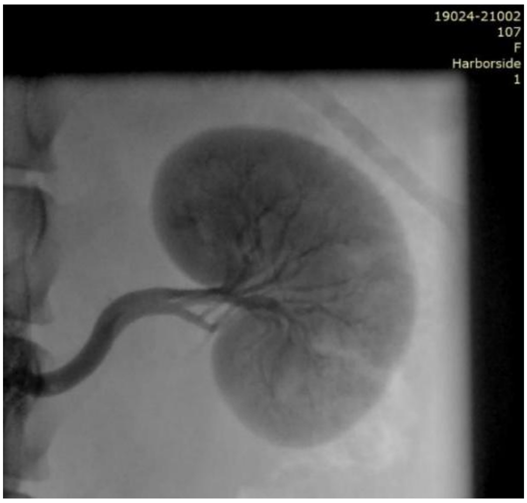


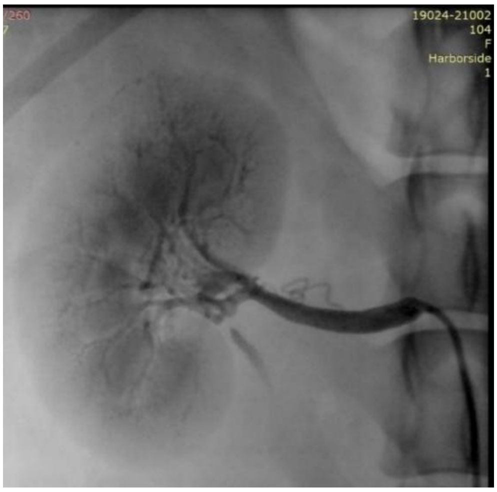


a. Left renal artery angiography(Animal 107 in the treatment group )

b. Right renal artery angiography(Animal 104 in the treatment group )

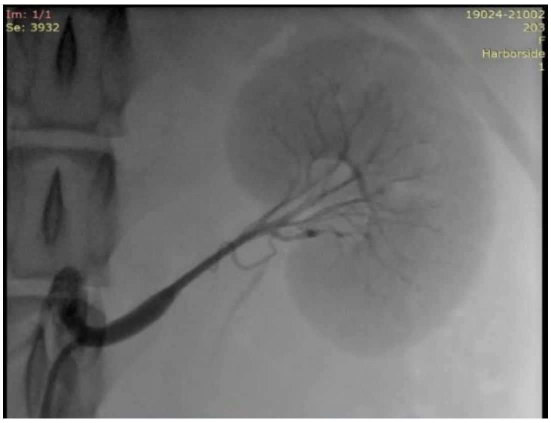


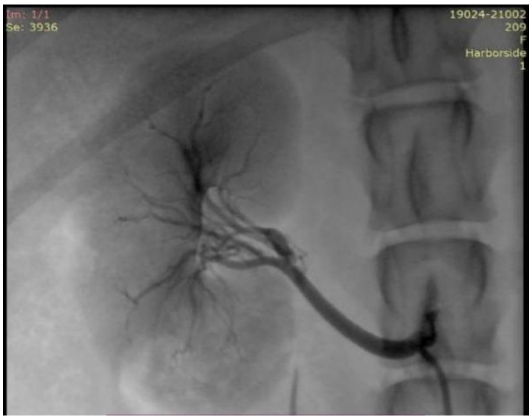


c.Left renal artery angiography(Animal 203 in the control group)

d. Right renal artery angiography(Animal 209 in the control group )

**Figure 7**

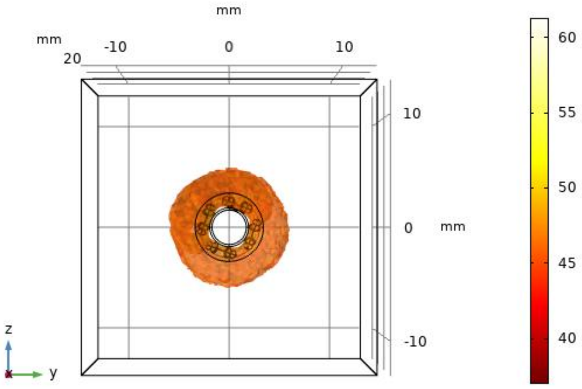


**Figure 8**

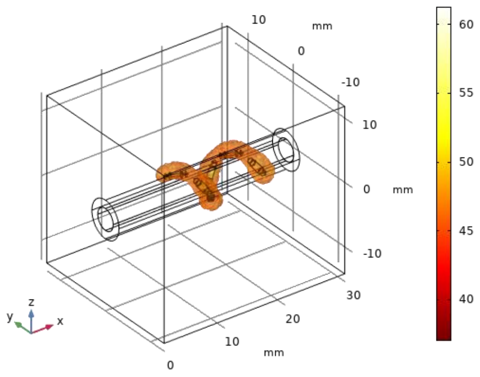


**Figure 9**

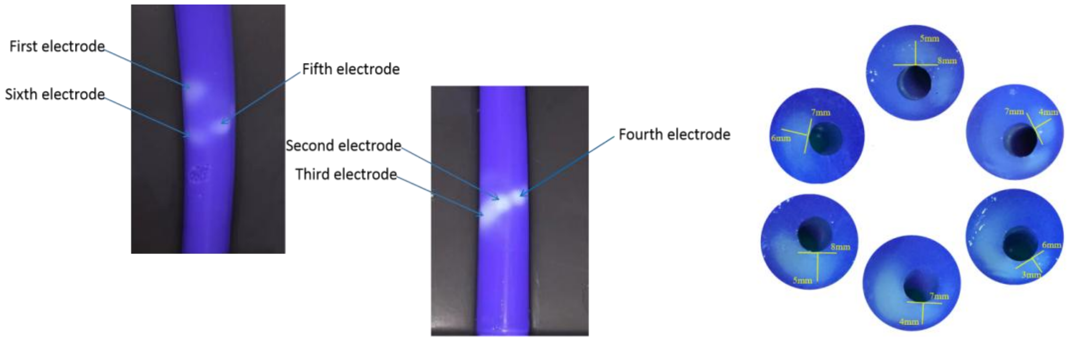


**Figure 10**

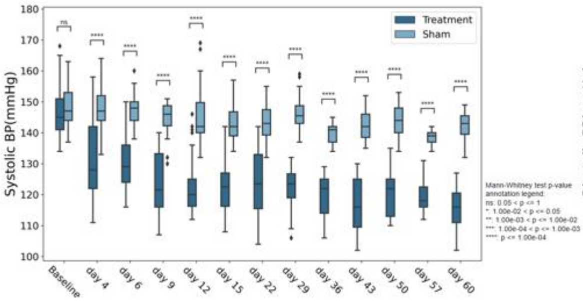


(a) Systolic BP

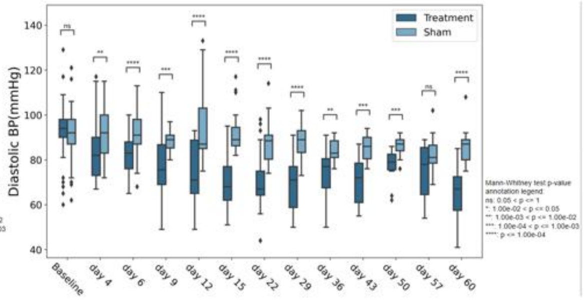


(b) Diastolic BP

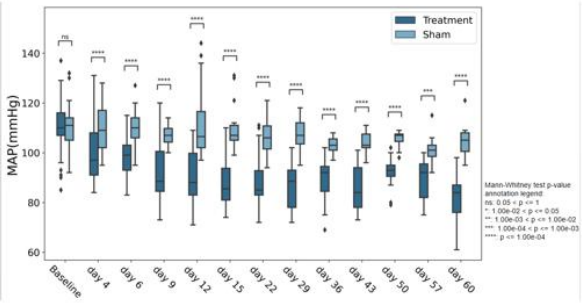


(c) MAP

**Figure 11**

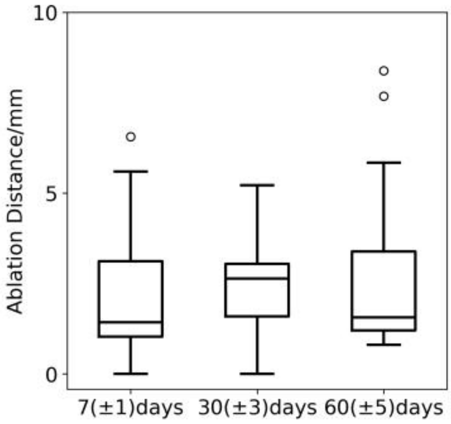


**Figure 12**

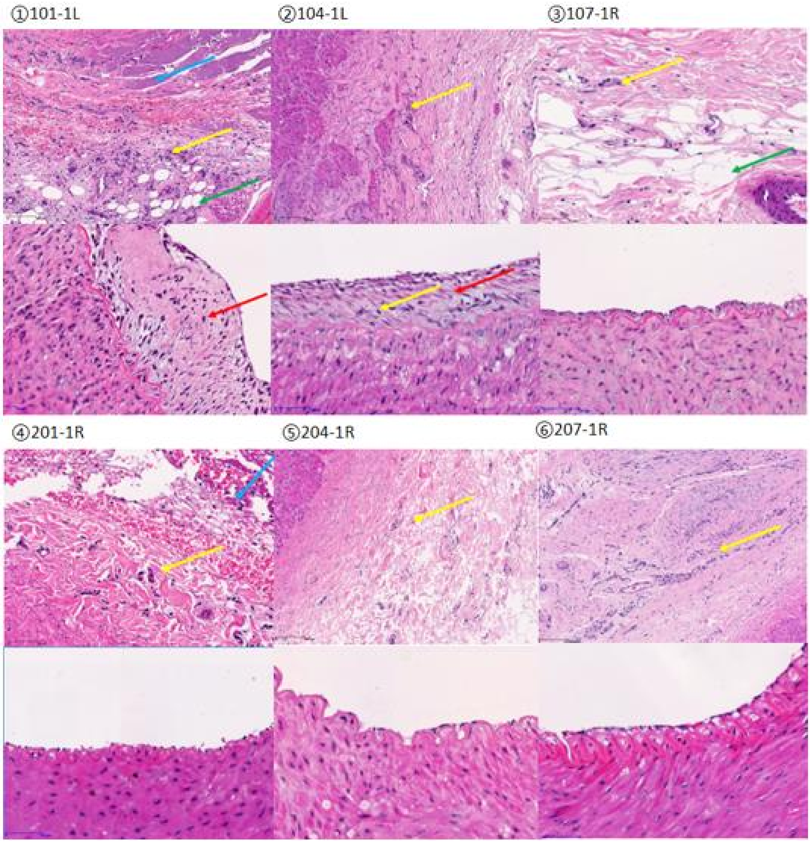


**Figure 13**

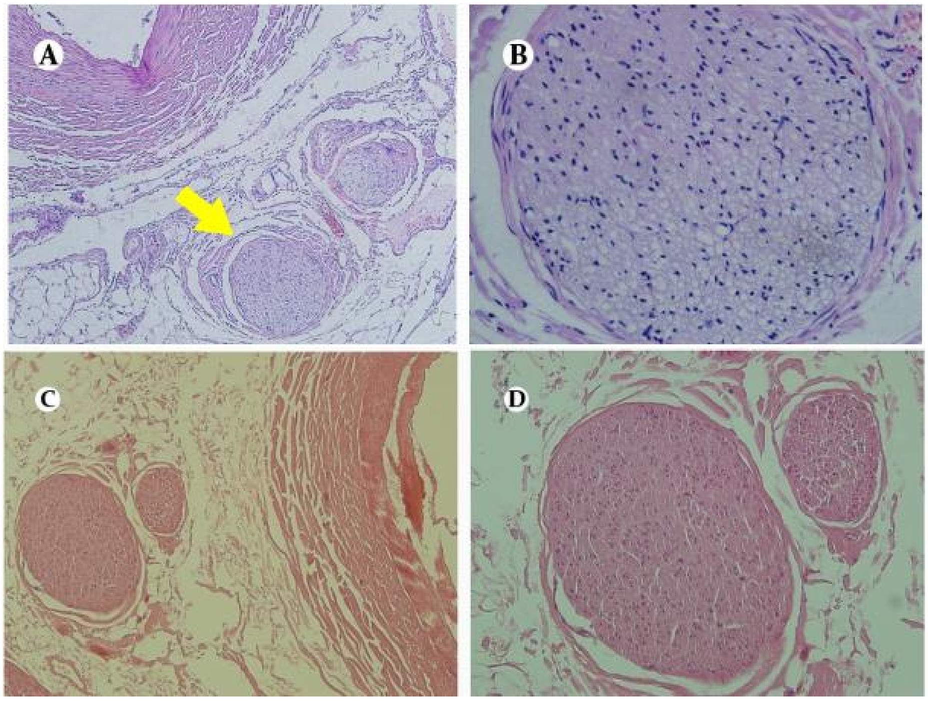


**Figure 14**

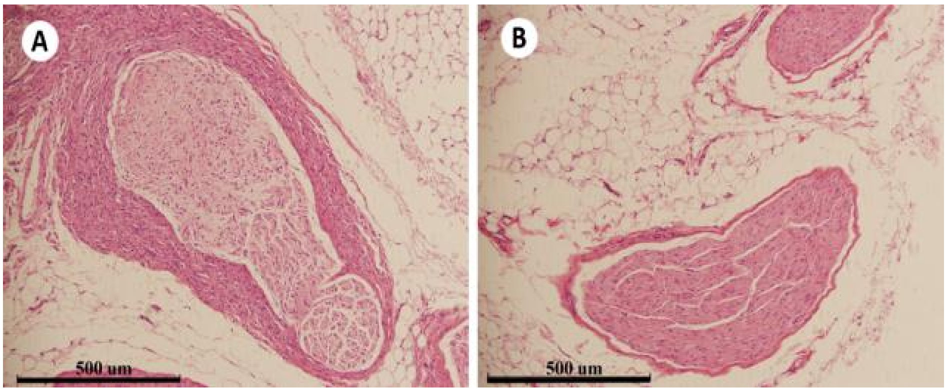


**Figure 15**

Click here to access/download
**Supporting Information**
Supporting Information-S1-raw-images.pptx

# Study on the efficacy of Netrod$^{TM}$ Six-electrode Radiofrequency Renal Denervation system on renal artery vessels

Xiaohua Song[ac1] Jiulin Guo[b1] Hongguang Cao[b] Qun Nan[a*]

[a] College of Chemistry and Life Science, Beijing University of Technology, Beijing, China

[b] Shanghai Golden Leaf MedTech CO., Ltd, Shanghai, China

[c] Industrial Informatization and Science and Technology Bureau of Changqing District, Jinan, China

**Abstract:** The principle of NetrodTM Renal Denervation RF Generator is based on the heating of radiofrequency electrodes to achieve the therapeutic temperature for peripheral nerve ablation with minimal impact on intra-arterial blood flow. The extravascular heat distribution and continuous ablation were evaluated using COMSOL Multiphysics with an idealized 3D model and validated in ex vivo experiment. The safety of ablation was verified by H&E staining of hypertensive dog model in treatment group of animal experiments, and changes in blood pressure before and after the procedure were used to characterize the efficacy. The simulation results showed that the maximum injury depth range of the Netrod$^{TM}$ Six-electrode is 5.08mm and the depth of the ablation zone obtained in the Phantom experiment was 3-6mm. Circumferential coverage was up to 91.99% and the ablation zone was continuously. In animal experiments, eight animals in the treatment group had significantly lower blood pressure (88.9%). Nerve cells in nerve bundles show vacuolar changes and thickening of peripheral sheaths after peripheral nerve ablation of renal arteries.The NetrodTM Six-electrode presented here has potential to effectively and safely ablate renal nerves. Systematic ablation can reach the target temperature in a shorter period of time, with faster warming, greater peripheral coverage of the lesion, and a more continuous ablation zone.

**Keywords:** Radio Frequency Ablation; Renal Denervation; Resistant hypertension; Temperature controlled; Netrod$^{TM}$ Six-electrode.

# Introduction

Hypertension is a cardiovascular syndrome with elevated arterial pressure in the body circulation as the main clinical manifestation and is an important risk factor for cardiovascular and cerebrovascular diseases. Hypertension is a common chronic disease that continues to increase in prevalence worldwide and is expected to reach 1.56 billion people by 2025, with refractory hypertension(RH) being particularly challenging to treat.[1]. Refractory hypertension(RH) is defined as hypertension in which blood pressure cannot be achieved even after adequate and reasonable combination of at least 3 antihypertensive drugs (including diuretics) based on dietary modification and lifestyle improvement[2]. RH accounts for about 5-15% of hypertensive patients, and some sources report that it can reach up to 20%-30%. There is still a clinical need for new easy, safe and effective treatments for hypertension.

Excessive sympathetic activation plays a significant role in the development and maintenance of hypertension [3-5]. Several studies have indicated that blocking sympathetic nerves may serve as a viable target for hypertension treatment [6-8]. Renal denervation (RDN) is a minimally invasive procedure that reduces blood pressure by modulating sympathetic hyperexcitability [9]. This technique is gaining acceptance and widespread application in clinical settings across various

* Corresponding author College of Chemistry and Life Science, Beijing University of Technology, Beijing, 100124, China
E-mail addresses: nanqun@ bjut. edu. cn
[1] Xiaohua Song and Jiulin Guo are Co-first authors

countries [10].Other studies[11] have also shown that RDN has the potential to extend to other autonomic nervous system disorders such as atrial fibrillation[12,13], heart failure[14,15] and tachycardia[16].

Although research related to RDN is still in its early stages, several new devices have been developed in recent years [17]. Common radiofrequency (RF) catheter ablation devices include the Symplicity System, Symplicity Spyral System, EnligHTN System, FlashPoint™ System, and Iberis System. We have developed the Netrod™ Six-electrode Radiofrequency Renal Denervation system and have investigated the safety and efficacy of the system in simulations, phantom experiment, and animal studies.

In the following sections, we will detail the numerical simulations and validation experiments conducted with the Netrod™ Six-electrode. This research aims to provide a scientific basis for assessing the clinical efficacy of renal sympathetic nerve ablation using the Netrod™ Six-electrode.

# Methods

## Equipment Introduction

Netrod™ Six-electrode Radiofrequency Renal Denervation system (Shanghai Golden Leaf MedTech Co. Ltd) includes Netrod™ Renal Denervation RF Generator and Netrod™ Six-electrode Radiofrequency Renal Denervation catheter (Fig 1).

[Here Fig. 1]

**Figure 1 Radiofrequency ablation mainframe and Netrod™ Six-electrode structure diagram**

The generator primarily consists of a microcontroller, a signal source circuit, a power drive circuit, a power output circuit, a detection circuit, and a display operation control unit. The detection circuit mainly detects monitors voltage, current, and temperature levels (Fig 2).

[Here Fig. 2]

**Figure 2 Netrod™ Renal Denervation RF Generator components**

The microcontroller is the core component of the generator. It not only processes the voltage, current, and temperature data transmitted by the detection circuit, but also outputs impedance, temperature, and power data to the display operation control unit. Furthermore, it intelligently adjusts the power output of the signal sources based on temperature and impedance to maintain the electrode surface temperature at a constant value, thereby ensuring safety during operation. The display operation control unit real-time records and displays the temperature, impedance, and power of each electrode during the radiofrequency ablation, and draws relevant curves (Fig 3), allowing medical staff to monitor the working status of the system in real-time through the display.

[Here Fig. 3]

**Figure 3 120 seconds of radiofrequency ablation, the power(P), temperature(T), and impedance(Z) of electrode 4 change over time**

The radiofrequency ablation catheter is composed of a central wire drawing with a developing head, a mesh tube connector, an electrode, a mesh basket bracket, a catheter sleeve, a seven-hole tube, and a handle assembly (Fig 4).

[Here Fig. 4]

**Figure 4 Netrod™ Six-electrode Basket Radiofrequency Renal Denervation Catheter. 1: Central wire drawing with a developing head; 2: Mesh basket bracket; 3: Electrode; 4: Seven-hole tube; 5: Adjustment knob; 6: Handle**

[Here Fig. 5]

**Figure 5 Basket catheter ablation of renal sympathetic nerves. A. Renal sympathetic nerve distribution; B,C. The basket catheter enters the branch for ablation; D. The basket**

**catheter expands the basket stent to perform ablation in the main trunk of the renal artery.**

# Finite element method modeling

We use COMSOL Multiphysics software (COMSOL, Burlington MA, USA), a finite element simulation software, to perform electromagnetic field and temperature field coupling calculations.

In radiofrequency ablation therapy, thermal energy is generated by converting the electrical energy that is applied as the application of radiofrequency current to the target renal artery tissue. The electric field control equation[18] can be calculated by solving the following Laplace equation. The frequency of the RF current is set to 470 KHz[19] , and the biological medium can be considered resistive because the displacement current is much smaller than the conduction current and thus can be neglected[20]. The distributed heat source Qr is given by Eq. (1)

$$Q_r = J \cdot E \tag{1}$$

Where J is the current density, measured in $A/m^2$; Qr is the distributed heat source, measured in J. The voltage is obtained from the solved Laplace equation, which is the controlling equation for solving the electric field problem in the presence of the internal power supply; see Eq. (2) and (3).

$$\nabla \cdot (\sigma \nabla V) = 0 \tag{2}$$

$$E = -\nabla V \tag{3}$$

Where V is the voltage, measured in V; E is the electric field strength, measured in V/m;$\sigma$ is the electrical conductivity, measured in S/m。In the model, the boundary conditions at the current interface are set as follows:

$$\begin{aligned} &V = 0 && \text{All external surfaces of the abdominal cavity} \\ &V = V_0 && \text{Six electrodes outer surface} \\ &\mathbf{n} \cdot J = 0 && \text{All other domain boundaries} \end{aligned} \tag{4}$$

The initial potential is set to 0 V, the outer surface of the electrodes is set to a voltage value with temperature control. In the entire model, all the outer surfaces of the tissue are grounded with zero current density.

In the simulation, an idealized three-dimensional vascular geometry model of the renal artery was constructed, as shown in Fig 6. The tissue included two major parts, the renal artery（6mm diameter) and the abdominal cavity (26mm×26mm×30mm), which was constructed as the external environment for the simulation. The arterial inlet flow rate was set to 0.5 m/s, and the ablation time was set to 120s.

[Here Fig. 6]

**Figure 6 Three-dimensional renal artery tissue and net basket-based RF electrode model**

**Table 1 shows material property parameters of the abdominal part, blood and electrodes**[21-25].

| Material Properties | ρ(kg/m$^3$) | σ (S/m) | C(J/kg·°C) | K(W/(m·°C) | $\xi_r$ |
|---|---|---|---|---|---|
| Blood | 1050 | 0.99 | 4180 | 0.54 | 58.3 |
| Abdominal cavity | 1060 | 1.69 | 3600 | 0.512 | 43.03 |
| Agar | 1300 | 2.3 | 4182 | 0.6 | 57.99 |
| Gold Electrode | 19300 | 4.1e7 | 127 | 315 | 1 |

Where ρ is the density, Kg/m$^3$; σ is the conductivity, S/m; C is the specific heat capacity, J/kg·°C; K is the thermal conductivity, m·°C; $\xi_r$ is the relative dielectric constant.

# Phantom experiment validation

Tissue-mimicking phantoms play a crucial role in the development, performance testing, quality assurance, and formulation of effective treatment plans for RF ablation devices[26]. Given that the walls of real human renal vessels are very thin, the wall thickness is often neglected in experimental models. In this study, we utilized a cylindrical mold with a vascular channel, where the surrounding area simulates human abdominal tissue. The entrance diameter of the vascular channel is 6 mm, corresponding to the dimensions of the simulation model. Polyacrylamide is particularly advantageous due to its stability at high temperatures. Many researchers have reported successful applications of polyacrylamide-based transparent tissue-mimicking molds, incorporating protein as a temperature indicator for thermal ablation studies[27]. The raw materials and formulation details for the specific mold used in this study are presented in Table 2.[28]

**Table 2 Proportioning table for the preparation of 100 ml solution**[29]

| Substance Name | Weighing |
|---|---|
| Citric acid | 2.49g |
| Sodium citrat | 2.66g |
| Acrylamide | 7.62g |
| Methylene-bisacrylamide | 0.4g |
| Bovine serum protein | 2.0g |
| Degassed water | 100ml |
| Ascorbic acid | 0.1g |
| 1%$FeSO_4$ | 1.0ml |
| 3%$H_2O_2$ | 1.0ml |

The target temperature for the electrode was set at 60˚C, and the experiment was conducted for a duration of 120 seconds. For thermal damage analysis, we evaluated the depth of thermal damage in the body mold through post-experimental assessment. The body mold changes color from blue to white when the temperature exceeds 45°C. Therefore, the color change on the surface of the body mold served as an indicator of the ablation effect.

# Animal study

Dogs subjected to animal testing and experimental protocols were treated in strict accordance approved by the Institutional Animal Care Committee of Shanghai Harborside Medical Technology Co., Ltd., based on the Institutional Animal Care and Use Committees（IACUC）Animal Care and Use Program on Animal Experimentation (approval No.IACUC-2021-003).

A total of 18 female experimental dogs (treatment group 101-109 and control group 201-209, 9 animals each) that had successfully established hypertension models were included in this experiment divided into three points of time, with 6 dogs in each point of time (3 animals each in the treatment group and the control group). The three points of time were 7 days (101-103 for the treatment group and 201-203 for the control group), 30 days (104-106 for the treatment group and 204-206 for the control group) and 60 days (107-109 for the treatment group and 207-209 for the control group). Under X-ray fluoroscopy, the left and right renal arteries of the animals in the treatment group were ablated with the system, and the operational performance of the test article was evaluated, and the Digital Subtraction Angiography(DSA) images are shown in Fig 7(a)-(d).

The duration of ablation was set to 120s. As a measure of efficacy, blood pressure was measured at regular intervals before and during the post-test observation period, and we measured the change in blood pressure at three points of time after RDN. During survival, animals were observed clinically. To investigate the short- term, medium- term, and long-term safety of the test article, three points of time, 7, 30, and 60 days, were included in this experiment. On postoperative days 7 (±1), 30 (±3) and 60 (±5) (with the day of surgery as 0 day), renal arteriograms were performed in animals scheduled for dissection to visualize the test site and renal arteries with nerves were harvested for immuno-histochemical evaluation. Animals were euthanized by deep anesthesia and an overdose of potassium chloride for autopsy and histopathological analysis. Quantification of protein denaturation and necrosis of renal sympathetic nerves around renal arteries were using hematoxylin-eosin (H&E) staining.

[Here Fig.7]

**Figure 7 Typical images of preanatomic renal arteriogram**

# Results

## Simulation results of Netrod™ Six-electrode

As the ablation time increases, the ablation area expands, with data detailed in Table 3.

**Table 3 Ablation of electrodes at different times**

| | Electrode temperature/˚C | Distance between 45˚C boundary and electrodes/mm |
|---|---|---|
| 5s | 48.6 | 0.23 |
| 30s | 60.0 | 2.69 |
| 60s | 60.0 | 3.78 |
| 120s | 60.0 | 5.08 |

With prolonged ablation time, the boundary of the region where the temperature reaches 45°C expands. Since temperature-controlled RF ablation was employed, the maximum temperature was confined to the vicinity of the electrodes, as shown in Table 3. The Netrod™ Six-electrode device can reach temperatures up to 45°C at distances of approximately 5.08 mm (>4 mm) from the electrode.

As shown in Fig 8， The Netrod™ Six-electrode can work simultaneously, and the mesh basket structure allows the electrodes to deliver RF energy close to the vessel wall.Netrod™ Six-electrodecan achieve 360° total ablation of sympathetic nerves in 30s.

[Here Fig.8]

**Figure 8 The effective ablation angle of Netrod™ Six-electrode(t=30s)**

The continuity of the Netrod™ Six-electrode ablation zone at an ablation time of 30s is shown in Fig 9. Each of the Netrod™ Six-electrode sits on a separate mesh basket of wires, which better conforms to the vessel wall as the lumen changes, keeping the electrode arrangement consistent.

[Here Fig.9]

**Figure 9 The continuity of the ablation area between the Netrod™ Six-electrode(t=30s)**

## Results of phantom experiments

As shown in Fig 10, during the ablation process, the six electrodes discharge to form a

continuous 360° spiral ribbon, with the ablation ranges of the first and sixth electrodes overlapping radially. The RF ablation experiments yielded resulted in an ablation zone with a width of 6-8 mm and a depth of 3-6 mm, as depicted in Fig 10.

The results from the phantom experiments validate the continuous ablation zone of the Netrod™ Six-electrode observed in the simulation. These experiments demonstrate that, despite the discrepancies in depth, the width of the ablation zone remains consistent, further supporting the accuracy of the simulation model. By comparing the body model experiments with the simulation results, we can confirm the effectiveness of the six electrodes in achieving a 360° spiral ablation. This consistency not only enhances confidence in the simulation model but also provides a reliable basis for clinical applications, indicating that the electrodes can effectively control ablation range and depth during actual procedures.

[Here Fig.10]

**Figure 10 Ablation effect of body mold experiment**

# Animal experiment results

The surgeon performed radiofrequency ablation of the left and right renal arteries of the hypertensive model animals according to the protocol (9 animals in the treatment group) or a sham procedure with renal artery angiography only (9 animals in the control group). There were no intraoperative abnormalities or serious adverse events related to animal vital signs, and the operability of the system was rated as excellent by the surgeon.

The group of the 18 experimental animals was mentioned in the previous section. The blood pressure of the animals was measured before and periodically after the procedure, and the blood pressure reduction data at different points of time are shown in Fig 11. Of the three animals in the test group at the point of time of 7 (±1) days, pre-anatomic diastolic and mean arterial pressures in animals 101 may not be statistically different relative to pre-ablation due to individual differences ($P>0.05$). In the other 2 animals, systolic, diastolic, and mean arterial pressure were significantly lower than before ablation ($P<0.05$). Blood pressure was significantly lower than before ablation ($P<0.05$) in all 6 animals in the test group at the 30 (±3) and 60 (±5) day point of time. Pre-ablation systolic, diastolic, and mean arterial pressures in the nine control animals during this procedure were only marginally reduced or increased relative to pre-sham procedure, with no significant differences ($P>0.05$).

[Here Fig.11]

**Figure 11 Values of blood pressure reduction in animals of the treatment group at different point of time**

The body weights of the experimental animals were not significantly abnormal during the experimental period, and the clinicopathological indices of the animals did not show any significant health-related abnormalities in the preoperative and pre-dissection test results. During the postoperative observation period, the experimental animals did not show any abnormalities related to the treatment. No abnormalities such as stenosis or dissection were observed in the renal arteries of the animals when the angiography was performed before dissection, and no abnormalities were observed when the animals were dissected for observation and sampling. The above experiments prove the safety of the device. The results of the validity analysis in the histopathological analysis showed that nerve fiber damage around bilateral renal artery vessels in animals of the treatment group at each point of time, showing different degrees of vacuolar degeneration, hyaline degeneration and coagulative necrosis. Degeneration of nerve fibers around the renal artery vessels was also seen in pathological section samples from most of the control animals, which was considered to be nerve injury reaction caused by the sham operation itself. In the histopathological

analysis, the data we measured to obtain the maximum distance from the ablation point to the nerve fiber damage in the treatment group are shown in Fig 12.

[Here Fig.12]

**Figure 12 Distance of nerve damage at different points of time**

The distance of nerve injury at the three points of time was not particularly different, and it was inferred that reversible repair of the injured nerve would not occur over time. The results of H&E staining of the ablation sites showed that the intima of the renal artery vessels in all groups of animals showed varying degrees of thickening and inflammatory infiltration. None of them showed desquamation, dissection, calcification or organization. Occasional there is thrombus formation in the lumen, which was considered to be caused by blood clotting in the blood vessels during animal dissection. Vascular injury and inflammatory infiltration of the tunica media were present to varying degrees in all groups of animals and no contractures or pseudoaneurysm were present. Occasional hyaline degeneration of the muscularis was seen in both groups, which was considered to be caused by ablation or the procedure itself. Different degrees of Necrosis of perivascular tissue, inflammatory infiltration, fibrosis and fatty infiltration were seen in the adventitia and perivascular tissue of renal arteries in both groups, and the overall pathological irritant response was more pronounced in the test group than in the blank control group. An overview of the efficacy and safety index pathological results is shown in Fig 13 for representative samples at different time periods, with animal number 101 (7 days), 104 (30 days) and 107 (60 days) for the treatment group and 201 (7 days), 204 (30 days) and 207 (60 days) for the blank control group.

[Here Fig.13]

**Figure 1 H&E staining of nerves (inflammatory cells shown by yellow arrows; tissue necrosis shown by blue arrows; fatty infiltration shown by green arrows; intimal thickening shown by red arrows)**

Figure 14A and B show the peripheral nerve of the renal artery after ablation, and a large number of vacuolar changes of nerve cells in the nerve bundle are seen; C and D are the peripheral nerve of the renal artery in the blank control group, and no significant abnormal changes were seen in the nerve bundles. The H&E staining pathological pictures of the canine renal artery peripheral nerve 42 days after the controlled experimental radiofrequency ablation of the renal artery are shown in Figure 15. The peripheral nerve bundle of the renal artery in the test group showed morphological changes and thickening of the peripheral sheath. No significant abnormal changes were seen in the peripheral nerve bundle of the renal artery in the blank control group. Combining the pathological results of the above efficacy and safety indicators, it was inferred that the treatment could effectively ablate perivascular nerve tissue and cause irreversible damage to nerve fibers. The degree of local inflammation and other pathological reactions in the treatment group was significantly greater than that in the blank control group at all points of time, which was considered to be caused by intraoperative ablation damage to tissues, but did not show significant safety problems, and all of them decreased with the prolongation of postoperative time.

[Here Fig.14]

**Figure 2 H&E staining of the peripheral nerve of the renal artery**

[Here Fig.15]

**Figure 3 H&E staining of the peripheral nerve of the renal artery after 42 days**

# Discussion

AI Raisi et al. [30] reported a circumferential coverage angle of 54.90° ± 4.36° for a single-electrode RF application, encompassing approximately 15% of the arterial wall circumference. In contrast, the circumferential coverage angle achieved by the Netrod™ Six-electrode was 360°. Yanyan Cheng et al. noted that the placement of spiral electrodes could cause blood to flow spirally

along the catheter, potentially resulting in more significant blood flow disturbances. Following balloon electrode placement in the renal artery, normal blood flow is obstructed, resulting in maximum blood flow velocity at the front of the electrode [31]. The mesh basket design of the Netrod™ Six-electrode effectively mitigates blood flow obstruction caused by both the spiral structure and the balloon.

The relevant parameters (including size and material) of the electrodes used in the ex vivo and animal experiments are the same as those of the electrodes used in the simulation. However, our preliminary research has some limitations. First, we utilized a simplified model of the Netrod™ Six-electrode and the renal arteries, omitting the full structure of the Netrod™ Six-electrode and only simulating the electrode positions. Although the electrode positions can vary and adhere to the vessel wall according to the lumen diameter, the contact force between the electrodes and tissue was not considered in our model. While simulation studies provide valuable insights, there remains a significant gap between these findings and their clinical application. In a clinical setting, factors such as patient-specific anatomy, variability in tissue properties, and real-time physiological responses can significantly influence the outcomes of RF ablation procedures. Additionally, the dynamic nature of blood flow and its interaction with the ablation device must be carefully assessed to optimize treatment effectiveness and minimize complications. Finally，the animal experiment were selected from 18 female dogs, and it was not possible to determine whether gender had an effect on the results of the experiment. And experimental dogs may not reflect the characteristics of human arteries with hypertension. Depth data on the canine model does not fully reflect ablation depths in other animals and in humans.

To bridge this gap, future studies should focus on integrating more complex and realistic models that account for these variables, including variations in blood flow dynamics and tissue response during ablation. Clinical trials will also be essential to validate the efficacy and safety of the Netrod™ Six-electrode in diverse patient populations demographics, ensuring that laboratory findings translate effectively into real-world applications.

# Conclusion

Through mathematical modeling, phantom experiments and animal study, we established a novel renal denervation system based on the Netrod™ Six-electrode. This system utilizes automatic temperature control and a mesh basket structure to target the nerves around the renal arteries. Simulation studies of the Netrod™ Six-electrode demonstrated that heat distribution effectively achieved denervation of the renal sympathetic nerves while maintaining a constant electrode temperature of 60°C.

This paper focuses on mathematical modeling, phantom experiments and animal studies to demonstrate the feasibility of the proposed new technique. We performed renal denervation in hypertensive dogs model and in an open surgical setting to show the efficacy and safety of the system in vivo. Further clinical studies are needed to validate the effectiveness and safety of the NetrodTM Six-electrode denervation system for blood pressure control in vivo.

# Data Availability Statement

All relevant data are within the paper.

# Competing interests

The authors have declared that no competing interests exist.

# Funding:

This work was supported by the National Natural Science Foundation of China [31771021,

11832003].The funder had role in study design, data collection and analysis.

# Author Contributions

Conceptualization: Xiaohua Song, Jiulin Guo, Hongguang Cao, Qun Nan

Data curation: Xiaohua Song, Jiulin Guo, Hongguang Cao, Qun Nan

Formal analysis: Xiaohua Song, Qun Nan

Writing-original draft: Xiaohua Song

Writing- review & editing: Xiaohua Song, Jiulin Guo, Hongguang Cao, Qun Nan

# References


[1] KEARNEY P M, WHELTON M, REYNOLDS K, et al. Global burden of hypertension: analysis of worldwide data [J]. Lancet, 2005, 365(9455): 217-23.

[2] CALHOUN D A, JONES D, TEXTOR S, et al. Resistant hypertension: diagnosis, evaluation, and treatment: a scientific statement from the American Heart Association Professional Education Committee of the Council for High Blood Pressure Research [J]. Circulation, 2008, 117(25): e510-26.

[3] YANG X, LIN L, ZHANG Z, et al. Effects of catheter-based renal denervation on renin-aldosterone system, catecholamines, and electrolytes: A systematic review and meta-analysis [J]. J Clin Hypertens (Greenwich), 2022, 24(12): 1537-1546.

[4] SHARP A S P, TUNEV S, SCHLAICH M, et al. Histological evidence supporting the durability of successful radiofrequency renal denervation in a normotensive porcine model [J]. J Hypertens, 2022, 40(10): 2068-2075.

[5] DíAZ-MORALES N, BARANDA-ALONSO E M, MARTíNEZ-SALGADO C, et al. Renal sympathetic activity: A key modulator of pressure natriuresis in hypertension [J]. Biochem Pharmacol, 2023, 208: 115386.

[6] ESLER M D, KRUM H, SCHLAICH M, et al. Renal sympathetic denervation for treatment of drug-resistant hypertension: one-year results from the Symplicity HTN-2 randomized, controlled trial [J]. Circulation, 2012, 126(25): 2976-2982.

[7] ESLER M D, BöHM M, SIEVERT H, et al. Catheter-based renal denervation for treatment of patients with treatment-resistant hypertension: 36 month results from the SYMPLICITY HTN-2 randomized clinical trial [J]. Eur Heart J, 2014, 35(26): 1752-1759.

[8] KRUM H, SCHLAICH M P, SOBOTKA P A, et al. Percutaneous renal denervation in patients with treatment-resistant hypertension: final 3-year report of the Symplicity HTN-1 study [J]. Lancet, 2014, 383(9917): 622-629.

[9] LINZ D, HOHL M, ELLIOTT A D, et al. Modulation of renal sympathetic innervation: recent insights beyond blood pressure control [J]. Clin Auton Res, 2018, 28(4): 375-384.

[10] GöTZINGER F, KUNZ M, LAUDER L, et al. Radio frequency-based renal denervation: a story of simplicity? [J]. Future Cardiol, 2023, 19(9): 431-440.

[11] GOYAL A, JAIN H, VERMA A, et al. The role of renal denervation in cardiology and beyond: An updated comprehensive review and future directives. Curr Probl Cardiol.2024 Feb;49(2):102196.

[12] ZUHAIR M ,KEENE D ,KANAGARATNAM P , et al.Percutaneous Neuromodulation for Atrial Fibrillation.[J].Cardiac electrophysiology clinics,2024,16(3):281-296.

[13] DOIRON E J ,LI Z ,YU X , et al.Early Renal Denervation Attenuates Cardiac Dysfunction in Heart Failure With Preserved Ejection Fraction.[J].Journal of the American Heart Association,2024,13(4):e032646-e032646.

[14] YING W ,MEIYAN S ,MEIFANG W , et al.Advances in device-based treatment of heart failure with preserved ejection fraction: evidence from clinical trials.[J].ESC heart failure,2023,11(1):13-27.

[15] LIANG X, SHANG S, BAI Z, et al. Effects of Renal Denervation on Gap Junction in High-Pacing-Induced Heart Failure Dogs. Anatol J Cardiol. 2024 Jul 16;28(9):429–36.

[16] TAKAHASHI K ,EGAMI Y ,NISHINO M , et al.Clinical impact of stellate ganglion phototherapy on ventricular tachycardia storm requiring mechanical circulatory support devices: a case report.[J].European heart journal. Case reports,2024,8(4):ytae177-ytae177.

[17] WHITE W B, GALIS Z S, HENEGAR J, et al. Renal denervation therapy for hypertension: pathways for moving development forward [J]. J Am Soc Hypertens, 2015, 9(5): 341-350.
[18] FANG Z, MOSER M A J, ZHANG E, et al. Design of a Novel Electrode of Radiofrequency Ablation for Large Tumors: In Vitro Validation and Evaluation [J]. J Biomech Eng, 2019, 141(3).
[19] POP M, MOLCKOVSKY A, CHIN L, et al. Changes in dielectric properties at 460 kHz of kidney and fat during heating: importance for radio-frequency thermal therapy [J]. Phys Med Biol, 2003, 48(15): 2509-2525.
[20] GONZáLEZ-SUáREZ A, BERJANO E. Comparative Analysis of Different Methods of Modeling the Thermal Effect of Circulating Blood Flow During RF Cardiac Ablation [J]. IEEE Trans Biomed Eng, 2016, 63(2): 250-259.
[21] PLOURDE B D, VALLEZ L J, SUN B, et al. The Effect of Plaque Removal on Pressure Drop and Flow Rate through an Idealized Stenotic Lesion [J]. Biology and Medicine, 2015, 8(1).
[22] Dong T，Cheng YY，Tian Z, et al. Ex-vivo experimental study in hepatic vascular to simulate the radiofrequency ablation of renal sympathetic nerve [J]. Beijing Biomedical Engineering, 2018, 37(03): 265-272+319.
[23] DONG T, NAN Q, TIAN Z, et al. Radiofrequency ablation of renal sympathetic nerve: Numerical simulation and ex vivo experiments [J]. Advances in Mechanical Engineering, 2018, 10(6).
[24] SHAO Y L, LEO H L, CHUA K J. Studying the thermal performance of a bipolar radiofrequency ablation with an improved electrode matrix system: In vitro experiments and modelling [J]. Applied Thermal Engineering, 2017, 116.
[25] ZHENG F, BING Z, MICHAEL M, et al. Design of a Novel Electrode of Radiofrequency Ablation for Large Tumors: A Finite Element Study [J]. Journal of Engineering and Science in Medical Diagnostics and Therapy, 2018, 1(1).
[26] NEGUSSIE A H, PARTANEN A, MIKHAIL A S, et al. Thermochromic tissue-mimicking phantom for optimisation of thermal tumour ablation [J]. Int J Hyperthermia, 2016, 32(3): 239-243.
[27] CHEN Y, YOUN P, FURGESON D Y. Thermo-targeted drug delivery of geldanamycin to hyperthermic tumor margins with diblock elastin-based biopolymers [J]. J Control Release, 2011, 155(2): 175-183.
[28] Huang Y, Hu B, Shen E. Experimental study of interaction between radiofrequency ablation and realtime temperature monitoring. Chinese Journal of Interventional Imaging and Therapy 2010;7(03):296-300.
[29] Zhang BL, Hu B, Kuang SL,Huang Y,Wu R,Jiang LX,et al. A tissue-mimicking phantom for visualizing coagulation temperature distribution at the same range of biological tissue during thermal ablation. Chinese Journal of Medical Imaging Technology 2007;(09):1405-1408.
[30] AL RAISI S I, POULIOPOULOS J, BARRY M T, et al. Renal Artery Branch Denervation: Evaluation of Lesion Characteristics Using a Thermochromic Liquid Crystal Phantom Model [J]. Heart Lung Circ, 2020, 29(3): 445-451.
[31] CHENG Y, LIU H, TIAN Z, et al. Evaluating the thermal performance of a balloon-based renal sympathetic denervation system with array electrodes: a finite element study [J]. Electromagn Biol Med, 2021, 40(4): 488-501.

# Response to Reviewers

## Journal Requirements:

1. Please ensure that your manuscript meets PLOS ONE's style requirements, including those for file naming.

Response: I have changed the format.

2. Please note that PLOS ONE has specific guidelines on code sharing for submissions in which author-generated code underpins the findings in the manuscript. In these cases, we expect all author-generated code to be made available without restrictions upon publication of the work.

Response:Yes, code will be made available upon publication of the work.

3. Thank you for stating the following financial disclosure:"This work was supported by the National Natural Science Foundation of China [31771021, 11832003]."Please state what role the funders took in the study. If the funders had no role, please state: ""The funders had no role in study design, data collection and analysis, decision to publish, or preparation of the manuscript."

Response: This work was supported by the National Natural Science Foundation of China [31771021, 11832003]. The funder had role in study design, data collection and analysis.

4. We note that your Data Availability Statement is currently as follows: All relevant data are within the manuscript and its Supporting Information files.

Response: Data Availability Statement is revised as follows: All relevant data are within the manuscript and its Supporting Information files.

5. Please amend either the abstract on the online submission form (via Edit Submission) or the abstract in the manuscript so that they are identical.

Response: I have modified the abstract to be consistent .

6. Please ensure that you refer to Figure 5 in your text as, if accepted, production will need this reference to link the reader to the figure.

Response: Figure 5 was drawn by us.

## Additional Editor Comments:

Additionally, in the first paragraph of Introduction, I would mention the clinical utility of renal denervation currently, beyond hypertension. You could refer to reviews encapsulating these findings, such as: https://doi.org/10.1016/j.cpcardiol.2023.102196.

Response: Thanks for the heads up on that.I added to the introduction the current state of research in hypertension other other clinical effects of renal denervation, etc.And cited the article as reference [11].

Content: Other studies[11] have also shown that RDN has the potential to extend to other

autonomic nervous system disorders such as atrial fibrillation[12,13], heart failure[14,15] and tachycardia[16].

Furthermore, the discussion requires work. An additional paragraph on comparing and contrasting your findings with existing literature, what's novel about your results, and some background with pros and cons on all three types of electrodes used and their comparisons in either the introduction or second paragraph of the discussion is warranted.
Response: Thank for your advice, I have made overall revisions to the content of the article. I focus the article on the simulation and experimental data of Netrod$^{TM}$ Six-electrode to verify the safety and effectiveness of this electrode through animal experimental data, and no longer compare the advantages and disadvantages with other types. Therefore, I reduced the description of other types of electrodes.

## Review Comments to the Author

## Reviewer 1:

1.In this manuscript, the authors examined the depth and circumference of ablation lesions produced by the Netrod and other radiofrequency renal denervation systems through simulation models and phantom experiments. While the information provided has clinical significance, there are several methodological limitations and content inconsistencies that cast doubt on the conclusions.
Response: Thank for your advice, in general I modified my research to focus on the experiments on this electrode. In this article, I have further refined and perfected the data from the animal study, and comprehensively demonstrated the efficacy and safety of the Netrod$^{TM}$ Six-electrode by using the data obtained from simulation, phantom experiments and the animal study. For details of the animal study, please refer to the section labeled "Animal study" (highlighted in red here), which records and analyzes the data (blood pressure changes and histological analysis) from the animal study in detail.

2.The title (referring to efficacy and safety, as shown on the first page, which differs from the title on the first page of the text) is inconsistent with the content presented, which only addresses the extent of ablations and does not discuss efficacy or safety.
Major limitations requiring clarification:
Response: Thank for your suggestion to add animal testing to verify its safety and efficacy. In the course of the experiment, through the monitoring of the animals' blood pressure, the stable performance of the Netrod$^{TM}$ Six-electrode in practical application was clearly demonstrated. Specifically, the experimental animals did not experience any abnormal physiological reactions or adverse reactions after receiving the electrode treatment, and all vital signs remained stable, which fully demonstrates that its safety can stand the test.The details are as follows:
Content: During the postoperative observation period, the experimental animals did not show any abnormalities related to the treatment. No abnormalities such as stenosis or dissection were observed in the renal arteries of the animals when the angiography was performed before dissection, and no abnormalities were observed when the animals were dissected for

observation and sampling. The above experiments prove the safety of the device.
3.Abstract Inconsistency: The abstract on the first page of the PDF file mentions animal studies and histological analysis. However, the main text and its abstract only present data on ablation lesion depth and circumference from different radiofrequency renal denervation systems. Additionally, the titles differ between sections. The content is limited and inconsistent with the title on the first page ("Study on the efficacy and safety of Netrod™ Six-electrode Radiofrequency Renal Denervation system on renal artery vessels"), as no information regarding clinical efficacy and safety is presented.
Response:Thank for your advice. I have refined the animal studies and histological analysis in the main text to validate the study on the efficacy of Netrod™ Six-electrode Radiofrequency through blood pressure changes in the animals and The results of H&E staining of the ablation sites. Renal Denervation system so that the abstract corresponds to the main text.At the same time I improved some of the article's headings to align them with the body content.

4. .Unsupported Assumptions in Introduction: The authors assume that "continuous circumferential (ring)" ablation is superior for renal denervation without providing supporting evidence. The Medtronic Symplicity Spyral system deliberately uses a "non-continuous" circumferential ablation pattern to prevent continuous vessel injury, which might lead to renal artery stenosis. The authors should provide histological evidence demonstrating the safety of continuous circumferential ablations achieved by the Netrod before asserting its superiority over non-continuous circumferential ablation in terms of efficacy or safety.
Response:As the reviewer noted, we do not discount the safety of Medtronic products in terms of continuous ablation, but only assessed the safety of continuous ablation with our own ablation electrodes in 18 animals. As a measure of efficacy, blood pressure was measured at regular intervals before and during the post-test observation period, and we measured the change in blood pressure at three points of time after RDN. During survival, animals were observed clinically. To investigate the short- term, medium- term, and long-term safety of the test article, three points of time, 7, 30, and 60 days, were included in this experiment. On postoperative days 7 (±1), 30 (±3) and 60 (±5) (with the day of surgery as 0 day), renal arteriograms were performed in animals scheduled for dissection to visualize the test site and renal arteries with nerves were harvested for immuno-histochemical evaluation. Quantification of protein denaturation and necrosis of renal sympathetic nerves around renal arteries were using hematoxylin-eosin (H&E) staining. Specific experimental results are shown in Animal experiment results. We believe that the design of the net basket electrodes allows multiple electrodes to contact the vessel simultaneously and create a continuous line of ablation at the desired location, which can improve the therapeutic efficacy.

5. Missing Table: Table 3 is referenced in Results 3.1 but is not present in the manuscript.
Response:Yes, thanks for the advice. The tables have been switched in content and location, and all tables have been sorted.

6. Incomplete Information in Figure 9: The vessel diameter in the simulation model should be specified, as this parameter significantly influences the continuity of ablation lesions across different systems.

Response:Yes, thanks for the advice. Figure 9 was removed because the focus was no longer on the helical electrode approach data. The diameter of the vessels in the simulation and in the soma experiments was 6 mm.There is a reference in “Finite element method modeling”:The tissue included two major parts, the renal artery（6mm diameter).

7. Inequitable System Comparisons: The default ablation times for different renal denervation systems are either 60 or 120 seconds. Comparing ablation extent at 30 seconds (Figure 8) or 5 seconds (Figure 9) provides an unfair comparison. Results should be shown for at least 60-second ablations across all systems.

Response:Yes, thanks for the advice. I agree with the reviewers that there was unfairness in the way the previous comparisons with other systems were made. Therefore, in this revision of the paper, instead of comparing other systems to highlight the advantages of the Netrod™ Six-electrode, we will focus on the presentation of the experimental data of the system itself. We will analyze the data obtained from simulations and in vivo experiments, and through detailed interpretation and analysis of these data, we will strongly argue efficacy and safety of the Netrod™ Six-electrode.

8.Contradictory Results: In Results 3.3, the authors note that phantom experiments allow for optimal manual positioning of the Netrod™ Six-electrode against the vessel wall, while simulation experiments show poorer wall apposition. However, the smaller ablation depth and extent observed in phantom experiments contradict the expected benefits of better contact. This discrepancy requires explanation.

Response:Thanks for the advice. It may be that our expression is not precise enough, we think: the simulation considers rigid walls or does not consider deformation, so the fit is worse than that of the phantom experiments , and therefore the depth is smaller. During the actual operation of the phantom experiments , adjustments can be made manually to make the mesh basket fit tightly, so the fit is better in phantom experiments, and the ablation depth is greater This may be the reason for the difference.

9.Incomplete Methodology: The ablation settings for other renal denervation systems are not described. These parameters should be clearly detailed to substantiate the fairness of the comparisons.

Response:Thanks for the advice. I agree with the reviewers that the previous comparisons with other systems were indeed unfair. Therefore, in this article, we will no longer use the comparison with other systems to highlight the advantages of theNetrod™ Six-electrode, but focus on the results of the experimental data of this system, with the help of concrete experimental data to strongly argue the rationality and effectiveness of this system, so that its advantages can be presented visually and more convincing.

## Reviewer 2:

1. The title of the manuscript does not indicate that this is an experiment and nor a clinical study.This is not even an animal study, but a mathematical simulation on software and phantoms that limits the overall relevance of the experiments.This should be specified in the

title and the conclusions of the manuscript.
Response:Yes, thank for your advice, and as a whole I've revised my research. The article was refined, mainly because the previous study was biased towards the mathematical simulation of the simulation and phantom experiments, but because of the lack of experimental data from animals or human beings, it limited the overall relevance of the study and did not well reflect the safety feasibility of the studied system, so the study increased the animal studies to further improve the experimental data on the basis of the mathematical simulation and match the simulation data to improve the study of this system.

2.First of all, this experimental study has not been performed in renal arteries, but in a cylindrical synthetic mold made of Polyacrylamide. Therefore, the title should not mention the words renal artery vessels but rather “polyacrylamide phantom” . Likewise, not been performed in biological tissues of living organisms it cannot refer to “safety” .Introduction. Authors should remove the comment on Flex and limit to spiral since the flex has no possibilities of spatial orientation of the electrode.
Response:Yes, thanks for the advice. I added animal testing to the study to demonstrate “safety” through changes in animal blood pressure data and histology.We did animal experiments, and the significant decrease in blood pressure in the animals after surgery and the histologic data after autopsy verified the effectiveness and safety (see Animal experiment results for details). And remove the comment on Flex and limit to spiral since the flex has no possibilities of spatial orientation of the electrode.

3.The Netrod$^{TM}$ Six-electrode device demonstrated effective ablation of renal sympathetic nerves, achieving temperatures of up to 45° C at distances of approximately 5.08 mm (>4 mm) from the electrodes. This confirms the feasibility and efficacy of using the NetrodTM Six-electrode for radiofrequency ablation of the renal sympathetic nerve” .Indeed, such a sentence cannot be accepted since by no means the authors are testing the effect of the device on renal nerves, but in a phantom. The reader would be mislead with such a sentence that should clearly state that was changes color as an indirect indicator of heat is a synthetic material and not real tissues.
Response:Yes, thanks for the advice. I think the reviewer made a reasonable point, and this formulation has been deleted, and to add in Phantom experiment validation:”The body mold changes color from blue to white when the temperature exceeds 45° C.”

4.To affirm that the observed changes obtained after heating the RF electrodes are effective in ablating nerves, the experiments should be performed in animal models or in human cadavers.
Response:Yes, thanks for the advice. The study has added animal experiments. Comparisons were made with the simulation to illustrate the accuracy of the simulation. Also blood pressure changes by animal experiments and renal arteriography was performed on animals scheduled for dissection to visualize the test site and renal arteries with nerves were collected for immunohistochemical evaluation.

5.Similarly, further in the results: “NetrodTM Six-electrode to be positioned optimally against the vessel wall” Or in the discussion: “Results show that the NetrodTM Six- electrode

system provides more comprehensive coverage and better continuity of the ablation zone compared to both the single electrode and the spiral six electrodes under the same setup. Lesion formation is influenced by several factors, including ablation power, ablation time, the number of electrodes, the area in contact with tissue, and blood flow"

Sentences as such cannot be proposed since there is no vessel wall, but a simple synthetic cylinder. Authors should therefore be clear with the reader and avoid any reference to tissues, vessels, blood and nerves.

Response:Yes, thank you for your suggestion. Instead of comparing the ablation zones of the two, we did a complete simulation, ex vivo and animal experiments for this electrode. So this part has been deleted.

6. Comparison of ablation effect of NetrodTM Six-electrode with single electrode and spiral six electrode: the authors do not explain the method used to detect and quantify the dispersion of the heath in figures 7,8 and 9.

Response:Yes, thanks for the advice. We will no longer compare the ablation zones of the different electrodes. Figure 7.8.9 has been changed so that the above issue no longer exists.

7.What is the model used to test 6 spiral electrodes? The commercially available spiral system (Medtronic) has 4 electrodes and not 6. If the spiral 6 electrodes used in this study was developed by the investigators please provide details of the model used and clarify that is not the one commercially available, and that the effect described for 6 electrodes may not apply to a 4 electrodes spiral catheter.

Response:Yes, thanks for the advice. Since the spiral electrode studied at the beginning of the article was based on the spiral electrode simulation of the spiral construction setup, without using the commercially available system, the study is not too rigorous, so the spiral system is no longer studied in this article, including the relevant simulation data.

8.Figure 10. Please explain how the phantom model was constructed and how was the effect of RF assessed.

Response:Yes, thanks for the advice. The model construction and materials are described in the Phantom experiment validation section. This model was used to assess the ablation depth of the device and the continuity of the ablation zone by the change in tissue color to validate the accuracy of the data from the simulation experiment.

9.The authors affirm that "The electrodes are treated as uniformly heated sources, spreading heat consistently into the bloodstream and surrounding tissue through conduction" how can they say that if their model has no fluid circulation, and therefore, temperature dispersion by blood temperature and flow cannot be calculated?

Response:Yes, thank you for your advice. The simulation takes into account the effect of blood flow, so there is fluid circulation, and it is mentioned in the text that "The arterial inlet flow rate was set to 0.5 m/s, and the ablation time was set to 120s." However, fluid circulation was not considered in the phantom due to the material structure and the specificity of the experiment.

10.Authors used 60° C for 120 seconds with their device.Other commercially available use variable temperature for 60 seconds. The authors should explain on which observations (ideally obtained in animals) they base the selection of temperature and duration of the energy delivery, and demonstrate in biological tissue of animals or cadavers, that such combination of temperature and duration is not creating unintended tissue damage to neighbor structures such as the artery wall, veins, lymphatic or urinary structures.

Response:Yes, thanks for the advice. The working time of the device is adjustable, and for the time case we added animal experiments, which further verified the safety and effecty of ablation at this time by animal experimental data (ablation time 120s, temperature control setting 60° C). It is not creating unintended tissue damage to neighbor structures such as the artery wall, veins, lymphatic or urinary structures.

Content:The degree of local inflammation and other pathological reactions in the treatment group was significantly greater than that in the blank control group at all points of time, which was considered to be caused by intraoperative ablation damage to tissues, but did not show significant safety problems, and all of them decreased with the prolongation of postoperative time.
