# Supplementary material for: The efficacy of Netrod Six-electrode Radiofrequency Renal Denervation system: mathematical modeling, phantom experiments and animal studies
Source: PLoS One. 2025 May 27;20(5):e0323336. doi: 10.1371/journal.pone.0323336 (PMC12111292; doi:10.1371/journal.pone.0323336)
Supplement: Attachment — Submitted filename: Response to Reviewers.docx [file pone.0323336.s001.docx]

Response to Reviewers

Journal Requirements:

1. Please ensure that your manuscript meets PLOS ONE's style requirements, including those for file naming.

 Response: I have changed the format.

2. Please note that PLOS ONE has specific guidelines on code sharing for submissions in which author-generated code underpins the findings in the manuscript. In these cases, we expect all author-generated code to be made available without restrictions upon publication of the work.

Response:Yes, code will be made available upon publication of the work.

3. Thank you for stating the following financial disclosure:"This work was supported by the National Natural Science Foundation of China [31771021, 11832003]."Please state what role the funders took in the study.  If the funders had no role, please state: ""The funders had no role in study design, data collection and analysis, decision to publish, or preparation of the manuscript."

Response: This work was supported by the National Natural Science Foundation of China [31771021, 11832003]. The funder had role in study design, data collection and analysis.

4. We note that your Data Availability Statement is currently as follows: All relevant data are within the manuscript and its Supporting Information files.

 Response: Data Availability Statement is revised as follows: All relevant data are within the manuscript and its Supporting Information files.

5. Please amend either the abstract on the online submission form (via Edit Submission) or the abstract in the manuscript so that they are identical.

 Response: I have modified the abstract to be consistent .

6. Please ensure that you refer to Figure 5 in your text as, if accepted, production will need this reference to link the reader to the figure.

 Response: Figure 5 was drawn by us.

Additional Editor Comments:

Additionally, in the first paragraph of Introduction, I would mention the clinical utility of renal denervation currently, beyond hypertension. You could refer to reviews encapsulating these findings, such as: https://doi.org/10.1016/j.cpcardiol.2023.102196.

Response: Thanks for the heads up on that.I added to the introduction the current state of research in hypertension other other clinical effects of renal denervation, etc.And cited the article as reference [11].

Content: Other studies[11] have also shown that RDN has the potential to extend to other autonomic nervous system disorders such as atrial fibrillation[12,13], heart failure[14,15] and tachycardia[16].

Furthermore, the discussion requires work. An additional paragraph on comparing and contrasting your findings with existing literature, what's novel about your results, and some background with pros and cons on all three types of electrodes used and their comparisons in either the introduction or second paragraph of the discussion is warranted.

Response: Thank for your advice, I have made overall revisions to the content of the article. I focus the article on the simulation and experimental data of Netrod^TM^ Six-electrode to verify the safety and effectiveness of this electrode through animal experimental data, and no longer compare the advantages and disadvantages with other types. Therefore, I reduced the description of other types of electrodes.

Review Comments to the Author

Reviewer 1:

1.In this manuscript, the authors examined the depth and circumference of ablation lesions produced by the Netrod and other radiofrequency renal denervation systems through simulation models and phantom experiments. While the information provided has clinical significance, there are several methodological limitations and content inconsistencies that cast doubt on the conclusions.

Response: Thank for your advice, in general I modified my research to focus on the experiments on this electrode. In this article, I have further refined and perfected the data from the animal study, and comprehensively demonstrated the efficacy and safety of the Netrod^TM^ Six-electrode by using the data obtained from simulation, phantom experiments and the animal study. For details of the animal study, please refer to the section labeled “Animal study” (highlighted in red here), which records and analyzes the data (blood pressure changes and histological analysis) from the animal study in detail.

2.The title (referring to efficacy and safety, as shown on the first page, which differs from the title on the first page of the text) is inconsistent with the content presented, which only addresses the extent of ablations and does not discuss efficacy or safety.

Major limitations requiring clarification:

Response: Thank for your suggestion to add animal testing to verify its safety and efficacy. In the course of the experiment, through the monitoring of the animals' blood pressure, the stable performance of the Netrod^TM^ Six-electrode in practical application was clearly demonstrated. Specifically, the experimental animals did not experience any abnormal physiological reactions or adverse reactions after receiving the electrode treatment, and all vital signs remained stable, which fully demonstrates that its safety can stand the test.The details are as follows:

Content: During the postoperative observation period, the experimental animals did not show any abnormalities related to the treatment. No abnormalities such as stenosis or dissection were observed in the renal arteries of the animals when the angiography was performed before dissection, and no abnormalities were observed when the animals were dissected for observation and sampling. The above experiments prove the safety of the device.

3.Abstract Inconsistency: The abstract on the first page of the PDF file mentions animal studies and histological analysis. However, the main text and its abstract only present data on ablation lesion depth and circumference from different radiofrequency renal denervation systems. Additionally, the titles differ between sections. The content is limited and inconsistent with the title on the first page ("Study on the efficacy and safety of Netrod™ Six-electrode Radiofrequency Renal Denervation system on renal artery vessels"), as no information regarding clinical efficacy and safety is presented.

Response:Thank for your advice. I have refined the animal studies and histological analysis in the main text to validate the study on the efficacy of Netrod^TM^ Six-electrode Radiofrequency through blood pressure changes in the animals and The results of H&E staining of the ablation sites. Renal Denervation system so that the abstract corresponds to the main text.At the same time I improved some of the article's headings to align them with the body content.

4. .Unsupported Assumptions in Introduction: The authors assume that "continuous circumferential (ring)" ablation is superior for renal denervation without providing supporting evidence. The Medtronic Symplicity Spyral system deliberately uses a "non-continuous" circumferential ablation pattern to prevent continuous vessel injury, which might lead to renal artery stenosis. The authors should provide histological evidence demonstrating the safety of continuous circumferential ablations achieved by the Netrod before asserting its superiority over non-continuous circumferential ablation in terms of efficacy or safety.

Response:As the reviewer noted, we do not discount the safety of Medtronic products in terms of continuous ablation, but only assessed the safety of continuous ablation with our own ablation electrodes in 18 animals. As a measure of efficacy, blood pressure was measured at regular intervals before and during the post-test observation period, and we measured the change in blood pressure at three points of time after RDN. During survival, animals were observed clinically. To investigate the short- term, medium- term, and long-term safety of the test article, three points of time, 7, 30, and 60 days, were included in this experiment. On postoperative days 7 (±1), 30 (±3) and 60 (±5) (with the day of surgery as 0 day), renal arteriograms were performed in animals scheduled for dissection to visualize the test site and renal arteries with nerves were harvested for immuno-histochemical evaluation. Quantification of protein denaturation and necrosis of renal sympathetic nerves around renal arteries were using hematoxylin-eosin (H&E) staining. Specific experimental results are shown in Animal experiment results. We believe that the design of the net basket electrodes allows multiple electrodes to contact the vessel simultaneously and create a continuous line of ablation at the desired location, which can improve the therapeutic efficacy.

5. Missing Table: Table 3 is referenced in Results 3.1 but is not present in the manuscript.

Response:Yes, thanks for the advice. The tables have been switched in content and location, and all tables have been sorted.

6. Incomplete Information in Figure 9: The vessel diameter in the simulation model should be specified, as this parameter significantly influences the continuity of ablation lesions across different systems.

Response:Yes, thanks for the advice. Figure 9 was removed because the focus was no longer on the helical electrode approach data. The diameter of the vessels in the simulation and in the soma experiments was 6 mm.There is a reference in “Finite element method modeling”:The tissue included two major parts, the renal artery（6mm diameter).

7. Inequitable System Comparisons: The default ablation times for different renal denervation systems are either 60 or 120 seconds. Comparing ablation extent at 30 seconds (Figure 8) or 5 seconds (Figure 9) provides an unfair comparison. Results should be shown for at least 60-second ablations across all systems.

Response:Yes, thanks for the advice. I agree with the reviewers that there was unfairness in the way the previous comparisons with other systems were made. Therefore, in this revision of the paper, instead of comparing other systems to highlight the advantages of the Netrod^TM^ Six-electrode, we will focus on the presentation of the experimental data of the system itself. We will analyze the data obtained from simulations and in vivo experiments, and through detailed interpretation and analysis of these data, we will strongly argue efficacy and safety of the Netrod^TM^ Six-electrode.

8.Contradictory Results: In Results 3.3, the authors note that phantom experiments allow for optimal manual positioning of the Netrod™ Six-electrode against the vessel wall, while simulation experiments show poorer wall apposition. However, the smaller ablation depth and extent observed in phantom experiments contradict the expected benefits of better contact. This discrepancy requires explanation.

Response:Thanks for the advice. It may be that our expression is not precise enough, we think: the simulation considers rigid walls or does not consider deformation, so the fit is worse than that of the phantom experiments , and therefore the depth is smaller. During the actual operation of the phantom experiments , adjustments can be made manually to make the mesh basket fit tightly, so the fit is better in phantom experiments, and the ablation depth is greater This may be the reason for the difference.

9.Incomplete Methodology: The ablation settings for other renal denervation systems are not described. These parameters should be clearly detailed to substantiate the fairness of the comparisons.

Response:Thanks for the advice. I agree with the reviewers that the previous comparisons with other systems were indeed unfair. Therefore, in this article, we will no longer use the comparison with other systems to highlight the advantages of theNetrod^TM^ Six-electrode, but focus on the results of the experimental data of this system, with the help of concrete experimental data to strongly argue the rationality and effectiveness of this system, so that its advantages can be presented visually and more convincing.

Reviewer 2:

1. The title of the manuscript does not indicate that this is an experiment and nor a clinical study.This is not even an animal study, but a mathematical simulation on software and phantoms that limits the overall relevance of the experiments.This should be specified in the title and the conclusions of the manuscript.

Response:Yes, thank for your advice, and as a whole I've revised my research. The article was refined, mainly because the previous study was biased towards the mathematical simulation of the simulation and phantom experiments, but because of the lack of experimental data from animals or human beings, it limited the overall relevance of the study and did not well reflect the safety feasibility of the studied system, so the study increased the animal studies to further improve the experimental data on the basis of the mathematical simulation and match the simulation data to improve the study of this system.

2.First of all, this experimental study has not been performed in renal arteries, but in a cylindrical synthetic mold made of Polyacrylamide. Therefore, the title should not mention the words renal artery vessels but rather “polyacrylamide phantom”. Likewise, not been performed in biological tissues of living organisms it cannot refer to “safety”.Introduction. Authors should remove the comment on Flex and limit to spiral since the flex has no possibilities of spatial orientation of the electrode.

Response:Yes, thanks for the advice. I added animal testing to the study to demonstrate “safety” through changes in animal blood pressure data and histology.We did animal experiments, and the significant decrease in blood pressure in the animals after surgery and the histologic data after autopsy verified the effectiveness and safety (see Animal experiment results for details). And remove the comment on Flex and limit to spiral since the flex has no possibilities of spatial orientation of the electrode.

3.The Netrod^TM^ Six-electrode device demonstrated effective ablation of renal sympathetic nerves, achieving temperatures of up to 45°C at distances of approximately 5.08 mm (>4 mm) from the electrodes. This confirms the feasibility and efficacy of using the NetrodTM Six-electrode for radiofrequency ablation of the renal sympathetic nerve”.Indeed, such a sentence cannot be accepted since by no means the authors are testing the effect of the device on renal nerves, but in a phantom. The reader would be mislead with such a sentence that should clearly state that was changes color as an indirect indicator of heat is a synthetic material and not real tissues.

Response:Yes, thanks for the advice. I think the reviewer made a reasonable point, and this formulation has been deleted, and to add in Phantom experiment validation:”The body mold changes color from blue to white when the temperature exceeds 45°C.”

4.To affirm that the observed changes obtained after heating the RF electrodes are effective in ablating nerves, the experiments should be performed in animal models or in human cadavers.

Response:Yes, thanks for the advice. The study has added animal experiments. Comparisons were made with the simulation to illustrate the accuracy of the simulation. Also blood pressure changes by animal experiments and renal arteriography was performed on animals scheduled for dissection to visualize the test site and renal arteries with nerves were collected for immunohistochemical evaluation.

5.Similarly, further in the results: “NetrodTM Six-electrode to be positioned optimally against the vessel wall”Or in the discussion: “Results show that the NetrodTM Six- electrode system provides more comprehensive coverage and better continuity of the ablation zone compared to both the single electrode and the spiral six electrodes under the same setup. Lesion formation is influenced by several factors, including ablation power, ablation time, the number of electrodes, the area in contact with tissue, and blood flow”

Sentences as such cannot be proposed since there is no vessel wall, but a simple synthetic cylinder. Authors should therefore be clear with the reader and avoid any reference to tissues, vessels, blood and nerves.

Response:Yes, thank you for your suggestion. Instead of comparing the ablation zones of the two, we did a complete simulation, ex vivo and animal experiments for this electrode. So this part has been deleted.

6. Comparison of ablation effect of NetrodTM Six-electrode with single electrode and spiral six electrode: the authors do not explain the method used to detect and quantify the dispersion of the heath in figures 7,8 and 9.

Response:Yes, thanks for the advice. We will no longer compare the ablation zones of the different electrodes. Figure 7.8.9 has been changed so that the above issue no longer exists.

7.What is the model used to test 6 spiral electrodes? The commercially available spiral system (Medtronic) has 4 electrodes and not 6. If the spiral 6 electrodes used in this study was developed by the investigators please provide details of the model used and clarify that is not the one commercially available, and that the effect described for 6 electrodes may not apply to a 4 electrodes spiral catheter.

Response:Yes, thanks for the advice. Since the spiral electrode studied at the beginning of the article was based on the spiral electrode simulation of the spiral construction setup, without using the commercially available system, the study is not too rigorous, so the spiral system is no longer studied in this article, including the relevant simulation data.

8.Figure 10. Please explain how the phantom model was constructed and how was the effect of RF assessed.

Response:Yes, thanks for the advice. The model construction and materials are described in the Phantom experiment validation section. This model was used to assess the ablation depth of the device and the continuity of the ablation zone by the change in tissue color to validate the accuracy of the data from the simulation experiment.

9.The authors affirm that “The electrodes are treated as uniformly heated sources, spreading heat consistently into the bloodstream and surrounding tissue through conduction” how can they say that if their model has no fluid circulation, and therefore, temperature dispersion by blood temperature and flow cannot be calculated?

Response:Yes, thank you for your advice. The simulation takes into account the effect of blood flow, so there is fluid circulation, and it is mentioned in the text that “The arterial inlet flow rate was set to 0.5 m/s, and the ablation time was set to 120s.”However, fluid circulation was not considered in the phantom due to the material structure and the specificity of the experiment.

10.Authors used 60°C for 120 seconds with their device.Other commercially available use variable temperature for 60 seconds. The authors should explain on which observations (ideally obtained in animals) they base the selection of temperature and duration of the energy delivery, and demonstrate in biological tissue of animals or cadavers, that such combination of temperature and duration is not creating unintended tissue damage to neighbor structures such as the artery wall, veins, lymphatic or urinary structures.

Response:Yes, thanks for the advice. The working time of the device is adjustable, and for the time case we added animal experiments, which further verified the safety and effecty of ablation at this time by animal experimental data (ablation time 120s, temperature control setting 60°C). It is not creating unintended tissue damage to neighbor structures such as the artery wall, veins, lymphatic or urinary structures.

Content:The degree of local inflammation and other pathological reactions in the treatment group was significantly greater than that in the blank control group at all points of time, which was considered to be caused by intraoperative ablation damage to tissues, but did not show significant safety problems, and all of them decreased with the prolongation of postoperative time.
