# Supplementary material for: The efficacy of Netrod Six-electrode Radiofrequency Renal Denervation system: mathematical modeling, phantom experiments and animal studies
Source: PLoS One. 2025 May 27;20(5):e0323336. doi: 10.1371/journal.pone.0323336 (PMC12111292; doi:10.1371/journal.pone.0323336)
Supplement: Attachment — Submitted filename: Response_to_Reviewers_auresp_2.docx [file pone.0323336.s003.docx]

Response to Reviewers

Journal Requirements:

1. Please review your reference list to ensure that it is complete and correct. If you have cited papers that have been retracted, please include the rationale for doing so in the manuscript text, or remove these references and replace them with relevant current references. Any changes to the reference list should be mentioned in the rebuttal letter that accompanies your revised manuscript. If you need to cite a retracted article, indicate the article’s retracted status in the References list and also include a citation and full reference for the retraction notice.

Response: Incomplete display of page numbers for reference[1] and incomplete journal titles for reference[3]. I've modified it.

Additional Editor Comments:

As I mentioned during the round one of revision. Please do add a more complete and expanded discussion. The discussion is too short and does not delve into comparing of your own results, with the results of existing literature on this topic that is published.

As I mentioned during the round one of revision. Please do add a more complete and expanded discussion. The discussion is too short and does not delve into comparing of your own results, with the results of existing literature on this topic that is published.

Please follow this format:

First paragraph of discussion: Key results

Second paragraph: Importance of your findings, and how it is clinically relevant.

Third paragraph: Comparison of your results with existing literature.

Fourth paragraph: Strengths and Limitations of your study

Response: Thank you for your suggestion. I have added the discussion section, which has been labeled in the original article.

Review Comments to the Author

Reviewer 1:

1.Title: The title “Study on the efficacy of NetrodTM Six-electrode Radiofrequency Renal Denervation system on renal artery vessels” does not match the findings. The authors provide information includes studies based on mathematical modeling, phantom experiments and animal study. It is not solely based on studies on renal arteries. It would be more rational and informative to revise the title to “The efficacy of NetrodTM Six-electrode Radiofrequency Renal Denervation system: mathematical modeling, phantom experiments and animal studies”..

Response: Thank you very much for your advice. I have revised the title.

2.Abstract: The statement “Systematic ablation can reach the target temperature in a shorter period of time, with faster warming, greater peripheral coverage of the lesion, and a more continuous ablation zone.” is not consistent with the findings. Given there is no comparison with other devices, it would not be rational to state that Netrod is “shorter”, “faster”, or “greater”. The authors should revise and describe the findings in a neutral manner.

Response: Thank you very much for your suggestion. In the absence of comparative data, it should not be generalized that these results are superior or inferior to other equipment.

3. Lesion continuity: Given that the depth of 45 degree boundary at 30 seconds of heating was 2.69 mm (Table 3), the distance between electrodes should be less than 5.4 mm to achieve continuity. The authors should introduce the distance between electrodes in the manuscript to make readers realize how continuity could be established, rather than simply showing a figure with no supportive data. Further, the authors did not provide data regarding the statement in the Abstract “Circumferential coverage was up to 91.99% and the ablation zone was continuously.” The authors should provide relevant data.

Response: Thank you very much for your advice. It is mentioned in the manuscript that based on the construction of the mesh basket-like electrodes of this system, the distance between the electrodes can be flexibly varied with the diameter of the blood vessels, and the distance between the electrodes is not a fixed value, so the distance between the electrodes is not explicitly mentioned in the manuscript. For continuity results, see the following and Figures 9 and 10.

Content: Each of the Netrod^TM^ Six-electrode sits on a separate mesh basket of wires, which better conforms to the vessel wall as the lumen changes, keeping the electrode arrangement consistent.

The continuity of the Netrod^TM^ Six-electrode ablation zone at an ablation time of 30s is shown in Fig 9. Each of the Netrod^TM^ Six-electrode sits on a separate mesh basket of wires, which better conforms to the vessel wall as the lumen changes, keeping the electrode arrangement consistent.

As shown in Fig 10, during the ablation process, the six electrodes discharge to form a continuous 360º spiral ribbon, with the ablation ranges of the first and sixth electrodes overlapping radially.

4.Lesion depth and width in animal studies: According to Figure 12, the mean ablation depth in animal studies seems to be less than 3 mm, which is less than the distance reported in simulation and phantom studies (5-6 mm). As the authors mentioned in the Discussion “AI Raisi et al. [30] reported a circumferential coverage angle of 54.90° ± 4.36° for a single electrode RF application, encompassing approximately 15% of the arterial wall circumference”, the authors should provide exact numbers of ablation depth and width (or coverage angle) in a Table and a representative H&E figure to show how the lesion depth and width were measured. The authors should discuss why there was such a discrepancy in ablation depth between animal studies and simulation studies and whether it would impact the so-called “continuity” to be established and the corresponding ablation strategy.

Response: Fig. 13 replaces Fig. 12, and the data in Fig. 13 are average data, some of which can reach the distance reported in the simulation and modeling studies (5-6 mm) because the data in the simulation and modeling studies were set up for an ideal state, whereas there is a gap between the depth of the simulation and the animal experiments due to the real arterial structure and arterial shape of the animals in the animal experiments. For the H&E graphs of the animal experiments, the initial intention in this study was to determine the effectiveness of this system through simulation, and to verify the safety of ablation through H&E staining of the hypertensive canine model, so the animal experiments did not go to validate the data of the simulation, but to further study that this system is safe, so it wasn't focused on, but the next step will be to go to get the accurate data through more animal experiments .

The discussion of animal experiments has been added as follows:

In this study, we verify the effectiveness of this system through modeling studies, and animal experiments are further to go to verify the effectiveness and safety of this system, as the data in the modeling studies are set up for an ideal state, and there is a gap between the depth and the animal experiments due to the real arterial structure of the animals and the shape of the arteries in the animal experiments.

5.Confusing terms and grammatical errors: It is confusing what “test article”, “preanatomic” (Figure 7 legend), “blank control” refer to. It might be “treatment group”, “pre-ablation” and “sham-control”, but it is definitely hard for readers. Extensive language editing is required.

Response: Thanks for the advice. The “test article” refers to “the experimental dogs of treatment group”. The “preanatomic” refer to “pre-dissection”. The “blank control” refer to “control group”.

I replaced “test article” with “the experimental dogs of treatment group” and replaced ”blank control” with ” control”.
